# Synthesis of ultra-high molecular weight poly(ethylene)-*co*-(1-hexene) copolymers through high-throughput catalyst screening†

**DOI:** 10.1039/d1ra00446h

**Published:** 2021-02-02

**Authors:** Thomas J. Williams, Jessica V. Lamb, Jean-Charles Buffet, Tossapol Khamnaen, Dermot O'Hare

**Affiliations:** Chemistry Research Laboratory, Department of Chemistry, University Oxford 12 Mansfield Road OX1 3TA Oxford UK dermot.ohare@chem.ox.ac.uk; SCG Chemicals Co., Ltd 1 Siam Cement Rd Bangkok 10800 Thailand

## Abstract

A family of permethylindenyl titanium constrained geometry complexes, Me_2_SB(^R′^N,^3-R^I*)TiX_2_ ((3-R-η^5^-C_9_Me_5_)Me_2_Si(^R′^TiX_2_)), supported on solid polymethylaluminoxane (sMAO) are investigated as slurry-phase catalysts for ethylene/H_2_ homopolymerisation and ethylene/1-hexene copolymerisation by high-throughput catalyst screening. Me_2_SB(^*t*Bu^N,I*)TiCl_2_ supported on sMAO [sMAO-Me_2_SB(^*t*Bu^N,I*)TiCl_2_] is responsive to small quantities of H_2_ (<1.6%), maintaining high polymerisation activities (up to 4900 kg_PE_ mol_Ti_^−1^ h^−1^ bar^−1^) and yielding polyethylenes with significantly decreased molecular weight (*M*_w_) (from 2700 to 41 kDa with 1.6% H_2_). In slurry-phase ethylene/1-hexene copolymerisation studies, a decrease in polymerisation activity and polymer molecular weights compared to ethylene homopolymerisation is observed. Compared to many solid supported system, these complexes all display high 1-hexene incorporation levels up to a maximum incorporation of 14.2 mol% for sMAO-Me_2_SB(^iPr^N,I*)TiCl_2_). We observe a proportionate increase in 1-hexene incorporation with concentration, highlighting the ability of these catalysts to controllably tune the amount of 1-hexene incorporated into the polymer chain to produce linear low-density polyethylene (LLDPE) materials.

## Abstract 2

Permethylindenyl titanium CGCs supported on solid polymethylaluminoxane (sMAO) are investigated as slurry-phase catalysts by high-throughput catalyst screening (copolymerisation of ethylene/1-hexene with incorporation levels up to 14.2 mol%).

## Graphical abstract

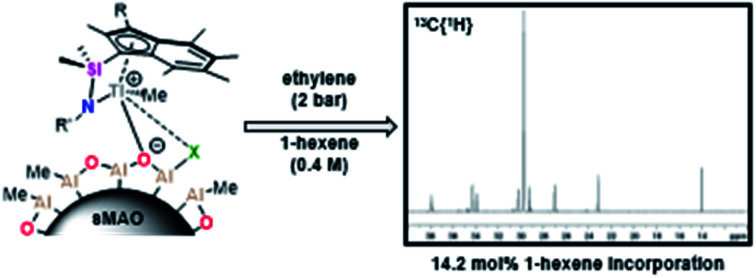

## Introduction

The incorporation of longer chain α-olefin monomers into polyethylene chains increases the degree of polymer branching, which lowers the melting point, crystallinity, and density of the polymers.^1^ This can lead to significant increases in polymer flexibility, which gives the resultant polymers applications in packaging, foams, elastic fibers, and adhesives.^2^

Metallocene catalysts containing two η^5^-cyclopentadienyl (C_5_H_5_, Cp) ligands and two σ-type ligands (Cp_2_MX_2_) have similar reactivities with both ethylene and longer chain α-olefins;^3^ allowing them to incorporate much larger percentages of higher α-olefins than traditional Ziegler–Natta catalysts.^4^ Unlike the latter, copolymerisation using metallocene catalysts often results in regular comonomer distributions and forms high strength, high clarity polymers.^5,6^

Constrained geometry complexes (CGCs), bridged half-metallocenes containing amide ligands, such as the Dow Chemical Co. complexes {(3-^*t*^Bu-η^5^-C_5_H_3_)Me_2_Si(^*t*Bu^N)}TiMe_2_ (Me_2_SB(^*t*Bu^N,Cp^3-*t*Bu^)TiMe_2_), Me_2_SB(^*t*Bu^N,Cp*)TiMe_2_, Me_2_SB(^*t*Bu^N,I)TiMe_2_, and Me_2_SB(^*t*Bu^N,^3-OMe^I)TiMe_2_,^7^ have been shown to be highly efficient ethylene/olefin copolymerisation catalysts, with high levels of olefin incorporated into the polymer chains.^5,8,9^ For example, in the solution phase, α-olefin incorporations of 25.3 mol% have been observed for ethylene/1-octene copolymerisation using Me_2_SB(^*t*Bu^N,Cp*)TiMe_2_/[HNMe(C_18_H_37_)_2_][B(C_6_F_5_)_4_] (20 bar ethylene and 300 g 1-octene),^7^ and incorporations of 69.9% for ethylene/1-hexene copolymerisation using Me_2_SB(^*t*Bu^N,Cp*)Ti(CH_2_Ph)_2_/MAO (1 bar ethylene and 44.5 mmol 1-hexene).^10,11^ These CGCs are of industrial interest due to their enhanced ability to copolymerise ethylene and longer chain α-olefins when compared to Cp_2_MX_2_ metallocene catalysts.^7,8,11,12^ This has been attributed to the less crowded coordination sphere, decreased tendency to undergo chain transfer reactions, and smaller bite angle (Cp_cent_–M–N angle) of CGCs compared to metallocenes (Cp_cent_–M–Cp_cent_) (by approximately 25–30°).^13^

CGCs are highly tuneable, and variation of the complex components can dramatically influence polymerisation activities.^13^ It has been found that for CGCs containing a substituted indenyl fragment, the addition of electron-donating substituents leads to both increased copolymerisation activity and polymer molecular weights.^14^ One advantage of CGCs is their ability to produce polyethylenes with very ultra-high molecular weights, with *M*_w_ often in excess of 1000 kDa.^10,15–17^ The long polymer chains transfer pressure more effectively to the polymer backbone, resulting in very tough materials with the highest impact strength of any thermoplastic currently produced.^17^ The extremely low moisture absorption, very low friction coefficient, biological inertness, and self-lubricating nature of UHMWPE have led to their use in fishing lines, joint replacements, and impact-resistant materials in the military.^17–19^

**Chart 1:**
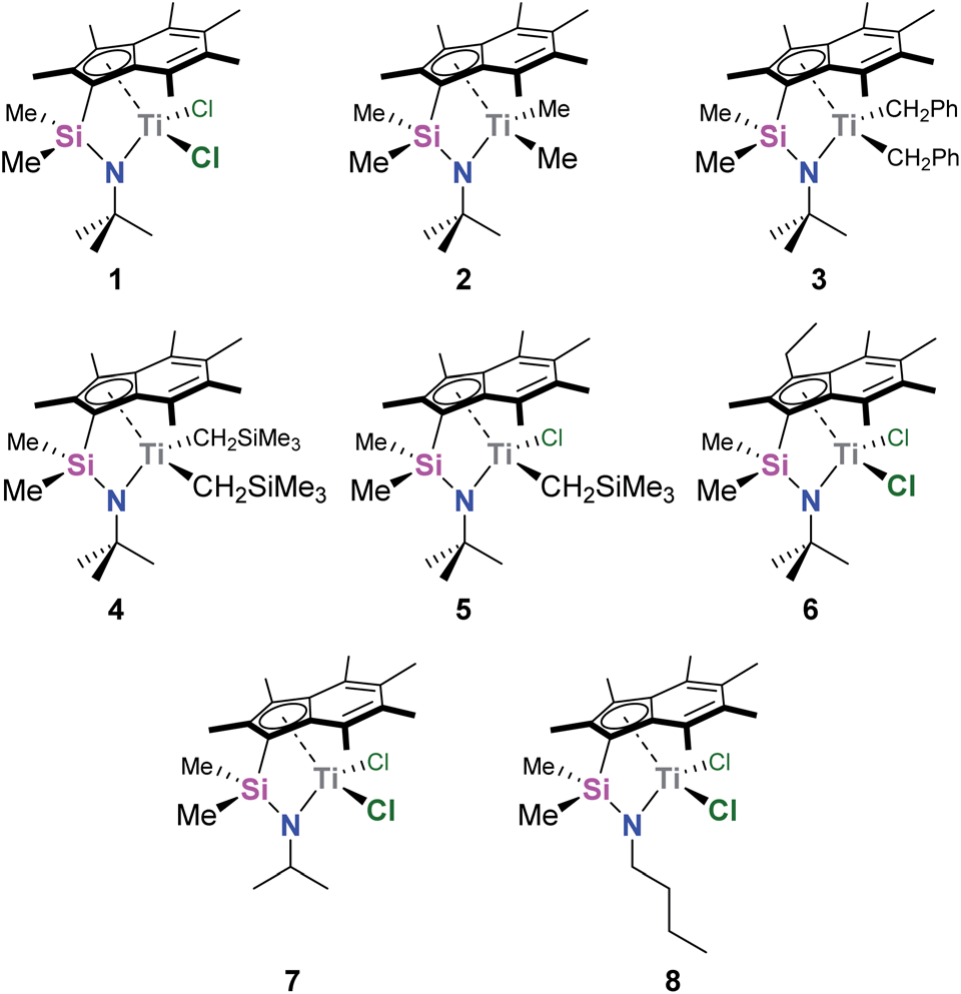
Permethylindenyl CGCs: Me_2_SB(^*t*Bu^N,I*)TiCl_2_ (1),^20^ Me_2_SB(^*t*Bu^N,I*)TiMe_2_ (2),^21^ Me_2_SB(^*t*Bu^N,I*)Ti(CH_2_Ph)_2_ (3),^21^ Me_2_SB(^*t*Bu^N,I*)Ti(CH_2_SiMe_3_)_2_ (4),^21^ Me_2_SB(^*t*Bu^N,I*)Ti(Cl)CH_2_SiMe_3_ (5),^21^ Me_2_SB(^*t*Bu^N,^3-Et^I*)TiCl_2_ (6),^21^ Me_2_SB(^iPr^N,I*)TiCl_2_ (7),^20^ and Me_2_SB(^*n*Bu^N,I*)TiCl_2_ (8).^20^

We recently reported the synthesis and characterisation of a new family of CGCs based on the permethylindenyl ligand (C_9_Me_7_, Ind*, I*): {(3-R-η^5^-C_9_Me_5_)Me_2_Si(^R′^N)}TiX_2_ (Me_2_SB(^R′^N,^3-R^I*)TiX_2_; R = H and Et; R′ = ^i^Pr, ^*t*^Bu, and ^*n*^Bu; X = Cl, Me, CH_2_Ph, and CH_2_SiMe_3_) (Chart 1).^20,21^

When immobilised on solid polymethylaluminoxane (sMAO),^22^ an insoluble form of oligomeric MAO, the CGCs were found to be very active catalysts for slurry-phase ethylene polymerisation, ethylene/1-hexene copolymerisation, and ethylene/styrene copolymerisation with activities up to 7048, 4248, and 2036 kg_PE_ mol_Ti_^−1^ h^−1^ bar^−1^ respectively.^21^ The catalysts showed low levels of 1-hexene and styrene incorporation (1.9–2.4 mol% and 1.6–2.5 mol% respectively) with 1-hexene incorporation levels found to increase with increasing copolymerisation temperature.^21^

Herein, we report a systematic investigation of the polymerisation performance of sMAO supported permethylindenyl titanium constrained geometry complexes for ethylene and ethylene/1-hexene copolymerisation using a high-throughput catalyst screening methodology.

## Results and discussion

The CGCs in Chart 1 were immobilised on solid polymethylaluminoxane (sMAO) with an initial aluminium to titanium catalyst loading ([Al_sMAO_]_0_/[Ti]_0_) of 200, using a procedure described in previous work.^20^ The catalysts were studied under high-throughput conditions for ethylene homopolymerisation with or without dihydrogen (H_2_), and ethylene/1-hexene copolymerisation. The high-throughput system allowed a large number of parallel experiments to be run simultaneously, enabling the screening of different conditions in a shorter time period.^23^

### Ethylene/H_2_ homopolymerisation

sMAO supported Me_2_SB(^*t*Bu^N,I*)TiCl_2_ (1_sMAO_), Me_2_SB(^*t*Bu^N,I*)TiMe_2_ (2_sMAO_), Me_2_SB(^*t*Bu^N,I*)Ti(CH_2_Ph)_2_ (3_sMAO_), Me_2_SB(^*t*Bu^N,I*)Ti(CH_2_SiMe_3_)_2_ (4_sMAO_), Me_2_SB(^*t*Bu^N,I*)Ti(Cl)CH_2_SiMe_3_ (5_sMAO_), and Me_2_SB(^*t*Bu^N,^3-Et^I*)TiCl_2_ (6_sMAO_) were studied for ethylene homopolymerisation and H_2_ response. High-throughput polymerisation studies were conducted in a parallel pressure reactor (PPR) at 80 °C with 8.3 bar ethylene, 0.8% (0.07 bar) or 1.6% (0.13 bar) H_2_ supplied by a mixed H_2_/N_2_ feed, 5 mL heptane, 10 μmol triisobutylaluminium (TiBA, Al(CH_2_CH(CH_3_)_2_)_3_) scavenger, and 0.075–0.40 mg pre-catalyst ([Al_sMAO_]_0_/[Ti]_0_ = 200) for 1 hour or until 8.3 bar of ethylene uptake was reached.

**Fig. 1:**
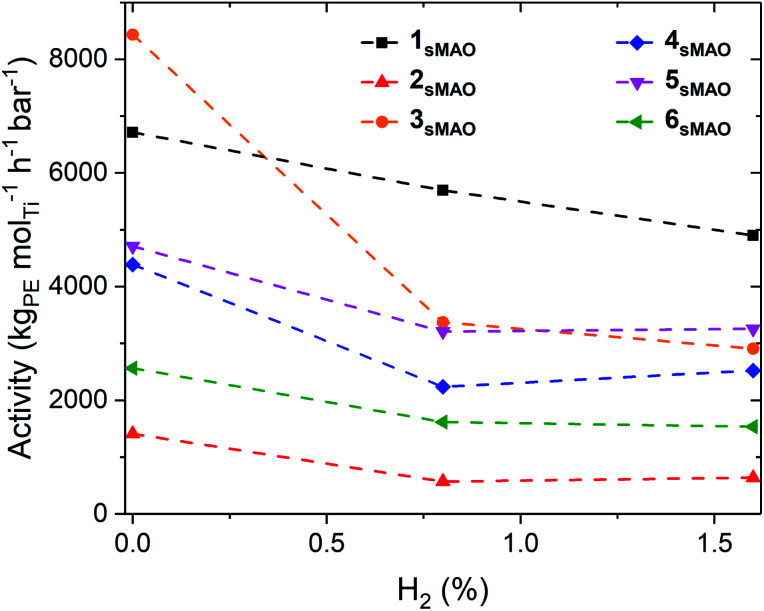
Slurry-phase ethylene polymerisation activity as a function of H_2_ (%) using sMAO supported Me_2_SB(^*t*Bu^N,I*)TiCl_2_ (1_sMAO_) (black square), Me_2_SB(^*t*Bu^N,I*)TiMe_2_ (2_sMAO_) (red up triangle), Me_2_SB(^*t*Bu^N,I*)Ti(CH_2_Ph)_2_ (3_sMAO_) (orange circle), Me_2_SB(^*t*Bu^N,I*)Ti(CH_2_SiMe_3_)_2_ (4_sMAO_) (blue diamond), Me_2_SB(^*t*Bu^N,I*)Ti(Cl)CH_2_SiMe_3_ (5_sMAO_) (pink down triangle), and Me_2_SB(^*t*Bu^N,^3-Et^I*)TiCl_2_ (6_sMAO_) (green left triangle) with 0, 0.8, and 1.6% H_2_. Polymerisation conditions: 8.3 bar ethylene, 0.075–0.40 mg pre-catalyst ([Al_sMAO_]_0_/[Ti]_0_ = 200), 5.0 mL heptane, 10 μmol TiBA, and 80 °C. Reactions quenched at 8.3 bar ethylene uptake or after 60 minutes.

Polymerisation activities decreased with the addition of H_2_, however, the catalysts remained very active; activities of 6700, 5700, and 4800 kg_PE_ mol_Ti_^−1^ h^−1^ bar^−1^ for 1_sMAO_ with 0, 0.8, and 1.6% H_2_ respectively (Fig. 1 and Table 1). The decrease in polymerisation activity with increasing H_2_ pressure was found to be greater for the alkylated catalysts (2_sMAO_, 3_sMAO_, and 4_sMAO_) than the dichloride (1_sMAO_ and 6_sMAO_) and mono-chloride (5_sMAO_) catalysts; with 1.6% H_2_, activity decreased by 28, 30, and 42% for 1_sMAO_, 5_sMAO_, and 6_sMAO_ when compared to ethylene homopolymerisation, and by 43, 54 and 65% for 4_sMAO_, 2_sMAO_, and 3_sMAO_. The differences in the relative changes in activities and the absolute activities of sMAO-Me_2_SB(^*t*Bu^N,I*)TiX_2_ (1_sMAO_–4_sMAO_) catalysts also suggests that the initiator groups remain coordinated to the surface of the support and influence the nature of the active species through a secondary coordination effect.^24^ Chlorides initiating group could also block the active sites.

**Table 1:** Slurry-phase ethylene/H_2_ polymerisation using sMAO supported I* CGCs in a high throughput system^a^

Catalyst	H_2_^b^	Activity^c^	*M*_w_ d	*M*_w_/*M*_n_
1_sMAO_	0	6700	2700	3.2
	0.8	5700	80	2.4
	1.6	4900	41	2.9
2_sMAO_	0	1400	1400	3.4
	0.8	570	85	2.7
	1.6	640	45	2.7
3_sMAO_	0	8400	1200	3.4
	0.8	3400	84	2.8
	1.6	2900	47	2.7
4_sMAO_	0	4400	1800	3.5
	0.8	2200	82	2.6
	1.6	2500	43	2.6
5_sMAO_	0	4700	1500	3.8
	0.8	3200	73	2.7
	1.6	3300	42	2.7
6_sMAO_	0	2600	1400	3.4
	0.8	1600	80	2.8
	1.6	1500	42	2.7

a Polymerisation conditions: 8.3 bar ethylene, 0.075–0.40 mg pre-catalyst ([Al_sMAO_]_0_/[Ti]_0_ = 200), 5.0 mL heptane, 10 μmol TiBA, and 80 °C. Reactions quenched at 8.3 bar ethylene uptake or after 60 minutes.

b %.

c kg_PE_ mol_Ti_^−1^ h^−1^ bar^−1^, reported to 2 significant figures.

d kDa, reported to 2 significant figures.

**Fig. 2:**
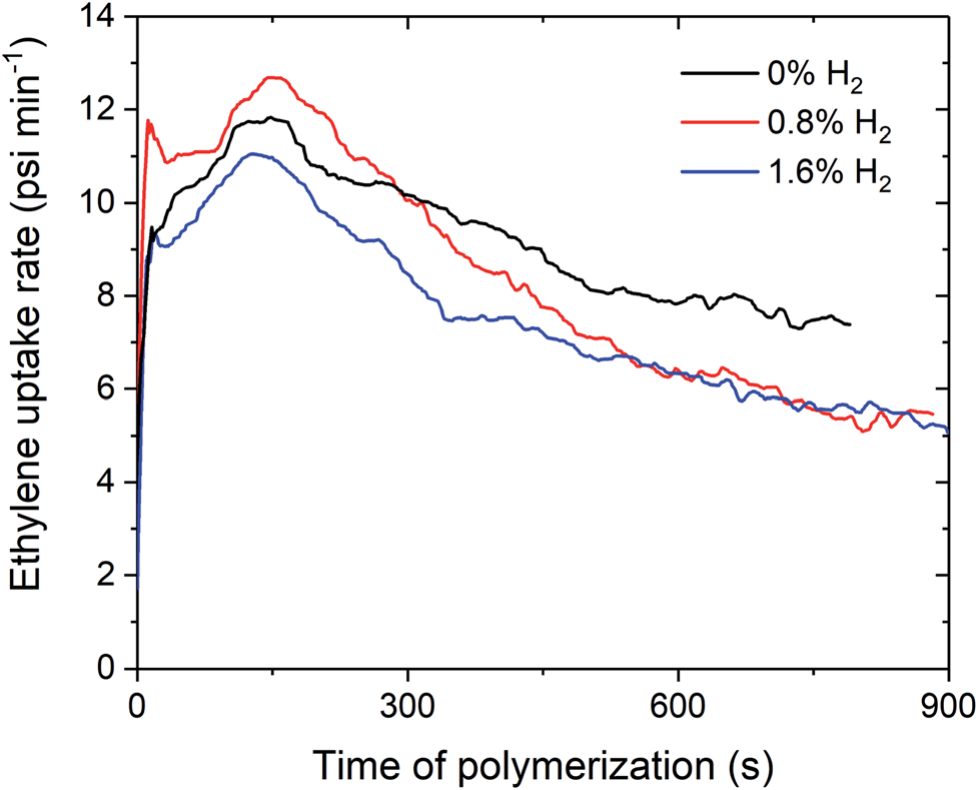
Slurry-phase polymerisation ethylene uptake rate as a function of time of polymerisation using sMAO supported Me_2_SB(^*t*Bu^N,I*)TiCl_2_ (1_sMAO_) with 0 (black), 0.8 (red), and 1.6% H_2_ (blue). Polymerisation conditions: 8.3 bar ethylene, 0.20 mg pre-catalyst ([Al_sMAO_]_0_/[Ti]_0_ = 200), 5.0 mL heptane, 10 μmol TiBA, and 80 °C. Reactions quenched at 8.3 bar ethylene uptake or after 60 minutes.

Over the course of the polymerisation runs, the *in situ* ethylene uptake rate profiles show lower uptake rates for ethylene polymerisation with H_2_ compared to without H_2_ (Fig. 2 and S1–S3†). The lower activities and ethylene uptake rates for ethylene/H_2_ polymerisation are attributable to the formation of a metal hydride species from chain transfer to H_2_, which requires reactivation by propagation.^25–27^ The lower polymerisation activities may also be due to the formation of dormant bimetallic resting states with a bridging hydride, as has been proposed in the solution phase, that require reactivation to form the cationic methyl species.^28,29^ For 1_sMAO_, ethylene polymerisation with 0.8% H_2_ initially shows a higher ethylene uptake rate than for polymerisation without H_2_; H_2_ may activate an alternative site for a short period,^27^ which then becomes deactivated as polymerisation progresses (Fig. 2).

Polymer molecular weights (*M*_w_) decreased with increased addition of H_2_; *M*_w_ of ∼80 and ∼45 kDa with 0.8 and 1.6% H_2_ respectively for all catalysts (Table 1, Fig. S9 and S11–S13†). The narrowing of the molecular weight distributions with increased addition of H_2_, (*M*_w_/*M*_n_ of 3.8 and 2.7 for 5_sMAO_ with 0 and 1.6% H_2_) suggests increased control in the reaction.^30^ Crystallisation-elution fractionation (CEF) showed that the maximum elution temperature (*T*_el,max_) of the polymers decreased slightly in the presence of H_2_ (*T*_el,max_ of 113.3, 112.1, and 111.8 °C with 0, 0.8, and 1.6% H_2_ respectively for 2_sMAO_), indicating a slight decrease in melting point and crystallinity (Table S1 and Fig. S18–S20†). The amorphous fraction (AF) increased in the presence of H_2_; AF of 0.2, 0.5 and 0.7 with 0, 0.8, and 1.6% H_2_ respectively for 2_sMAO_.

### Ethylene/1-hexene copolymerisation

sMAO supported Me_2_SB(^*t*Bu^N,I*)TiCl_2_ (1_sMAO_), Me_2_SB(^*t*Bu^N,I*)TiMe_2_ (2_sMAO_), Me_2_SB(^*t*Bu^N,I*)Ti(CH_2_Ph)_2_ (3_sMAO_), Me_2_SB(^*t*Bu^N,I*)Ti(CH_2_SiMe_3_)_2_ (4_sMAO_), Me_2_SB(^*t*Bu^N,I*)Ti(Cl)CH_2_SiMe_3_ (5_sMAO_), Me_2_SB(^*t*Bu^N,3-EtI*)TiCl_2_ (6_sMAO_), Me_2_SB(^iPr^N,I*)TiCl_2_ (7_sMAO_), and Me_2_SB(^*n*Bu^N,I*)TiCl_2_ (8_sMAO_) (Chart 1) were studied as catalysts for ethylene/1-hexene copolymerisation.

**Fig. 3:**
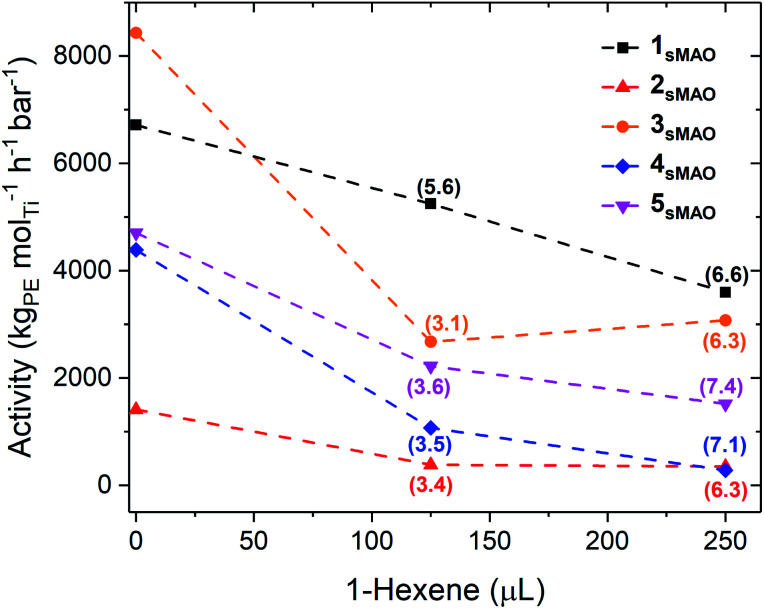
Slurry-phase ethylene polymerisation activity as a function of 1-hexene (μL) using sMAO supported Me_2_SB(^*t*Bu^N,I*)TiCl_2_ (1_sMAO_) (black square), Me_2_SB(^*t*Bu^N,I*)TiMe_2_ (2_sMAO_) (red up triangle), Me_2_SB(^*t*Bu^N,I*)Ti(CH_2_Ph)_2_ (3_sMAO_) (orange circle), Me_2_SB(^*t*Bu^N,I*)Ti(CH_2_SiMe_3_)_2_ (4_sMAO_) (blue diamond), and Me_2_SB(^*t*Bu^N,I*)Ti(Cl)CH_2_SiMe_3_ (5_sMAO_) (pink down triangle) with 0, 125, and 250 μL 1-hexene. 1-Hexene incorporation (mol%) shown in parenthesis. Polymerisation conditions: 8.3 bar ethylene, 0.075–0.40 mg pre-catalyst ([Al_sMAO_]_0_/[Ti]_0_ = 200), 5.0 mL heptane, 10 μmol TiBA, and 80 °C. Reactions quenched at 5.5 bar ethylene uptake or after 60 minutes.

Large reductions in activity were observed for ethylene/1-hexene copolymerisation compared to ethylene homopolymerisation (6700 and 3600 kg_PE_ mol_Ti_^−1^ h^−1^ bar^−1^ for 1_sMAO_ with 0 and 250 μL 1-hexene respectively), indicating that the negative comonomer effects outweigh the positive effects (Table 2, Fig. 3 and S8†).^21,31,32^ A large decrease in ethylene polymerisation activity is observed with increasing volumes of 1-hexene. For example, a decrease from 4700 to 1500 kg_PE_ mol_Ti_^−1^ h^−1^ bar^−1^ for 5_sMAO_ with 0 and 250 μL 1-hexene respectively.

**Table 2:** Slurry-phase ethylene/1-hexene copolymerisation using sMAO supported I* CGCs in a high-throughput system^a^

Catalyst	1-Hexene^b^	Activity^c^	*M*_w_ d	*M*_w_/*M*_n_	Incorporation^e^	*T*_el,max_ f
1_sMAO_	0	6700	2700	3.2	—	112.1
	125	5200	270	3.0	5.6	85.1
	250	3600	330	2.5	6.6	73.5
2_sMAO_	0	1400	1400	3.4	—	113.3
	125	380	390	2.8	3.4	89.0
	250	250	300	2.6	6.3	70.5
3_sMAO_	0	8400	1200	3.4	—	111.4
	125	2700	490	2.7	3.1	89.8
	250	3000	380	2.4	6.3	71.6
4_sMAO_	0	4400	1800	3.5	—	113.3
	125	1100	390	2.8	3.5	84.8
	250	280	200	2.3	7.1	69.5
5_sMAO_	0	4700	1500	3.8	—	113.3
	125	2200	360	2.5	3.6	88.0
	250	1500	270	2.5	7.4	70.9
6_sMAO_	0	2600	1400	4.0	—	113.4
	125	1700	250	2.9	5.6	81.9
	250	1100	220	2.6	8.4	66.0
7_sMAO_	0	1200	1700	2.5	—	113.9
	125	380	250	2.7	6.3	98.8
	250	390	260	2.4	14.2	80.5
8_sMAO_	0	490	1700	6.5	—	—
	125	220	210	4.3	1.6	—
	250	190	360	3.9	4.7	—

a Polymerisation conditions: 8.3 bar ethylene, 0.075–0.40 mg pre-catalyst ([Al_sMAO_]_0_/[Ti]_0_ = 200), 5.0 mL heptane, 10 μmol TiBA, and 80 °C. Reactions quenched at 5.5 bar ethylene uptake or after 60 minutes.

b μL.

c kg_PE_ mol_Ti_^−1^ h^−1^ bar^−1^, reported to 2 significant figures.

d kDa, reported to 2 significant figures.

e Mol%.

f °C.

Many theories have been proposed for the positive comonomer effect, including fracturing of catalyst particles exposing new sites, the formation of new active species by coordination of α-olefins, and activation of dormant active sites; however, many of these have been refuted for molecular catalyst systems.^33^ Studies have also shown that the addition of 1-hexene to an alkane reaction mixture leads to a 7–10% increase in ethylene solubility between 70–90 °C,^34^ as well as improved diffusion of ethylene close to the catalytic site, which improves polymerisation activity.^35^ The negative effects of comonomer addition are proposed to be due to competitive binding between ethylene and α-olefins and, if the rate of migratory insertion of the α-olefin is slower than that of ethylene, the rate of chain propagation will decrease leading to a decrease in polymerisation activity.^33^ The negative effects of comonomers on ethylene polymerisation activity may also be due to slower rates of insertion; the increased steric bulk of α-olefin comonomers in the polymer chain can lead to reduced rates of ethylene insertion.^36^

Through monitoring changes in temperature during polymerisation, an exothermic temperature spike to approximately 85 °C was observed at the start of the copolymerisation experiments. As the alkyl catalysts (2_sMAO_, 3_sMAO_, and 4_sMAO_) are much more sensitive to polymerisation temperature than the dichloride catalysts (1_sMAO_, 6_sMAO_, 7_sMAO_, and 8_sMAO_),^21^ this thermal spike caused more substantial decreases in polymerisation activities for these catalysts; activity decreases from 6700 to 3600 kg_PE_ mol_Ti_^−1^ h^−1^ bar^−1^ for 1_sMAO_ and from 4400 to 280 kg_PE_ mol_Ti_^−1^ h^−1^ bar^−1^ for 4_sMAO_ with 0 and 250 μL 1-hexene respectively.

**Fig. 4:**
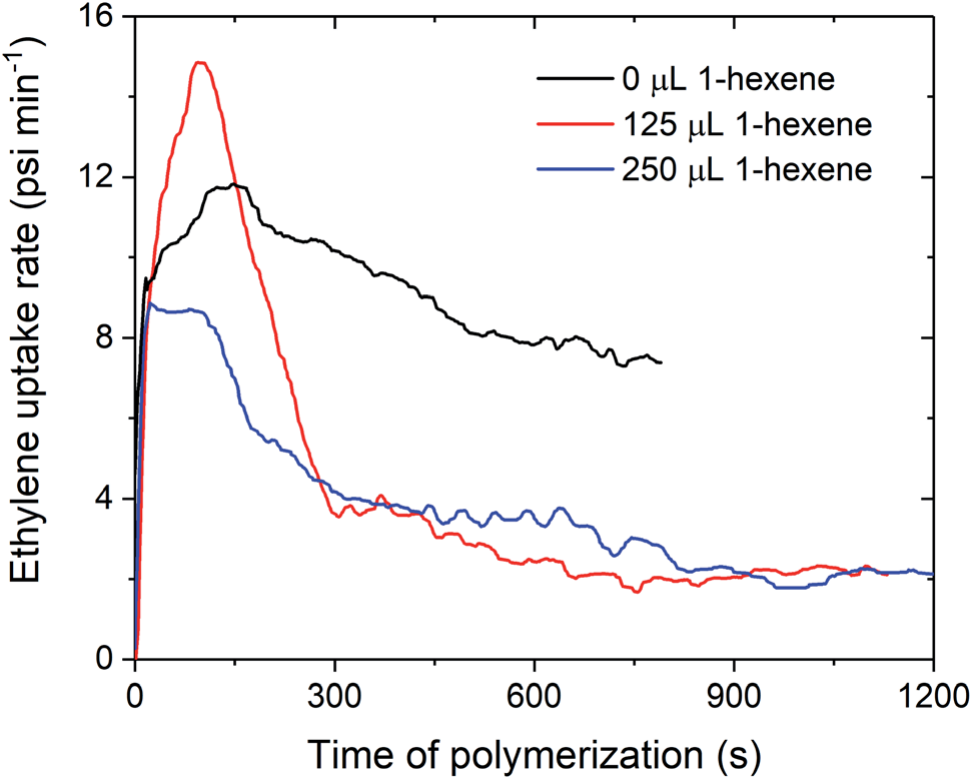
Slurry-phase polymerisation ethylene uptake rate as a function of time of polymerisation using sMAO supported Me_2_SB(^*t*Bu^N,I*)TiCl_2_ (1_sMAO_) with 0 (black), 125 (red), and 250 μL 1-hexene (blue). Polymerisation conditions: 8.3 bar ethylene, 0.20 mg pre-catalyst ([Al_sMAO_]_0_/[Ti]_0_ = 200), 5.0 mL heptane, 10 μmol TiBA, and 80 °C. Reactions quenched at 5.5 bar ethylene uptake or after 60 minutes.

The decreases in polymerisation activity with increasing volumes of 1-hexene are highlighted by the *in situ* ethylene uptake rate profiles, where sharp decreases in uptake rates with 125 and 250 μL 1-hexene are observed when compared to ethylene homopolymerisation (Fig. 4 and S4–S7†). Polymerisation activity was observed to increase with increasing electron-donating ability of the amido fragment (^*t*^Bu > ^i^Pr > ^*n*^Bu; 1_sMAO_ > 7_sMAO_ > 8_sMAO_) (Fig. S8†), as observed in previous work.^20^ Kamigaito *et al.* and Nomura *et al.* have also observed similar effects when using Me_2_SB(^R^N,Cp*)TiCl_2_/MAO (R = ^*t*^Bu, Ph, and C_6_F_5_;^37^ R = ^*t*^Bu and Cy)^38,39^ catalysts for solution-phase ethylene/styrene copolymerisation.

Klosin *et al.* have previously reported the effects of variation of the indenyl moiety on ethylene/1-octene copolymerisation, finding that increased electron-donating ability led to higher activities and polymer molecular weights.^14^ The opposite effect is observed for these systems, where 6_sMAO_ shows a lower ethylene polymerisation activity than 1_sMAO_, attributed to its decreased thermal stability; activities of 1100 and 3600 kg_PE_ mol_Ti_^−1^ h^−1^ bar^−1^ respectively with 250 μL 1-hexene. The lower polymerisation activity of 3-ethylpentamethylindenyl supported catalysts relative to the permethylindenyl analogs has been observed previously for ethylene polymerisation using 1_sMAO_ and 6_sMAO_ with 2 bar ethylene and 50 mL solvent at temperatures above 70 °C,^21^ and when using sMAO-Me_2_SB(2,7-^*t*Bu^Flu,^3-R^I*)ZrCl_2_ catalysts.^40^6_sMAO_ also shows greater decreases in activities for ethylene/1-hexene copolymerisation compared to ethylene homopolymerisation (35 and 58% decreases for 125 and 250 μL 1-hexene respectively) than 1_sMAO_ (22 and 46% decreases respectively). Similar to alkylated catalysts (2_sMAO_, 3_sMAO_, and 4_sMAO_), this may be due to the exothermic temperature spike at the beginning of the copolymerisation experiment and the lower thermal stability of 6_sMAO_ compared to 1_sMAO_.

The catalysts produced polymers with very high levels of 1-hexene incorporation for supported systems (up to 14.2 mol% for 7_sMAO_), confirming the production of ethylene/1-hexene copolymers. This is a trait commonly observed for CGCs that is attributed to the open metal centre resulting from the strain-inducing *ansa*-bridge (Table 2).^8,12,13^

The incorporation levels observed for these catalysts are lower than for solution-phase ethylene/1-hexene copolymerisation using Me_2_SB(^*t*Bu^N,Cp*)Ti(CH_2_Ph)_2_ with an MAO cocatalyst (65–70% 1-hexene incorporation).^10^ However, supported catalysts typically give lower incorporation levels than homogeneous catalysts due to mass transfer effects, where both the support and the propagating polymer chain cause diffusional resistance of the comonomer towards the active sites.^41,42^ The active sites of supported catalysts may also become blocked with polymer more quickly than the same catalysts in solution and therefore become inaccessible.^42^

It was found that 1_sMAO_, 6_sMAO_, and 7_sMAO_ produced polymers with similar incorporation levels with 125 μL 1-hexene (5.6–6.3 mol%). However, 7_sMAO_ produced polymers with much higher incorporation levels than 6_sMAO_ and 1_sMAO_ with 250 μL 1-hexene (14.2, 8.4, and 6.6 mol% respectively). This suggests that higher levels of 1-hexene incorporation accompany reduced steric bulk in the amido substituent, likely due to easier coordination of 1-hexene to the metal centre. Catalysts containing at least one alkyl ligand (2_sMAO_, 3_sMAO_, 4_sMAO_, and 5_sMAO_) produced polymers with similar incorporation levels; 3.1–3.6 and 3.3–7.4 mol% with 125 and 250 μL 1-hexene respectively.

A similar effect was also observed by Chen and Marks for solution-phase ethylene/1-hexene copolymerisation using Me_2_SB(^*t*Bu^N,Cp*)TiMe_2_/(BC_6_F_5_)_3_ and Me_2_SB(^*t*Bu^N,Cp*)Ti(CH_2_Ph)_2_/MAO where both alkyl ligand containing catalysts produced polymers with ∼70% 1-hexene incorporation.^10^8_sMAO_ consistently produced polymers with lower incorporation levels (1.6 and 4.7 mol% with 125 and 250 μL 1-hexene respectively), which may be due to the reduced electron donating ability of ^*n*^Bu. A proportionate increase in 1-hexene incorporation was observed for ethylene/1-hexene copolymerisation using 2_sMAO_–7_sMAO_ (the amount of 1-hexene incorporated into the polyethylene chain approximately doubled when the amount of 1-hexene in the system was doubled), which gives great potential for these catalysts to controllably tune the amount of 1-hexene incorporated into the polymer chain.

Gel permeation chromatography (GPC) showed that as the amount of 1-hexene added to the system increased, the molecular weights (*M*_w_) of the polymers significantly decreased; the polymers produced using 1_sMAO_ showed an eight-fold decrease in polymer molecular weights on the addition of 250 μL 1-hexene (*M*_w_ of 2700 and 330 kDa with 0 and 250 μL 1-hexene respectively) (Table 2, Fig. S10 and S14–S17†). The decrease in polymer molecular weights likely results from frequent chain termination following 1-hexene insertion and chain transfer to 1-hexene monomers, coupled with a decrease in the rate of chain propagation.^31,43^ This effect has been observed and studied for ethylene/α-olefin polymerisation using other CGC systems, such as Me_2_SB(^*t*Bu^N,Cp*)TiMe_2_, Me_2_SB(^*t*Bu^N,^2-R^I)TiMe_2_, and Me_2_SB(^*t*Bu^N,^3-R^I)TiMe_2_, with work having been undertaken in an attempt to negate the molecular weights decrease by adding heteroatom substituents in the 2- and 3-positions on the indenyl moiety.^7,14^

The catalysts produced polymers with relatively narrow molecular weight distributions (*M*_w_/*M*_n_), which became narrower with increasing volumes of 1-hexene; *M*_w_/*M*_n_ of 3.2, 3.0, and 2.7 for 1_sMAO_ with 0, 125, and 250 μL 1-hexeneThe polymers produced using 8_sMAO_ showed wider molecular weight distributions than the polymers produced using the other catalysts (*M*_w_/*M*_n_ of 6.5, 4.4, and 4.0 with 0, 125, and 250 μL 1-hexene respectively), suggesting the potential for more than one active site (Fig. S17†).

CEF showed that the maximum elution temperatures (*T*_el,max_) of the polymers dramatically decreased with increasing volumes of 1-hexene, indicative of higher levels of 1-hexene incorporation; *T*_el,max_ of 112.1, 85.1, and 73.5 °C for 1_sMAO_ with 0, 125, and 250 μL 1-hexene respectively (Table 2 and Fig. S21–S23†).

The decreases in *T*_el,max_ are attributable to the weakening of intramolecular forces between the polymer chains with increasing incorporation of 1-hexene and decreasing molecular weights of the polymers.^44^ The amorphous fraction (AF) also increased with increasing 1-hexene concentration; AF of 0.2, 0.7, and 27.2 wt% for 4_sMAO_ with 0, 125, and 250 μL 1-hexene respectively (Table S2†). This corroborates with the high temperature ^13^C{^1^H} NMR spectra (Fig. S24–S27†).

## Conclusions

A series of eight permethylindenyl constrained geometry titanium complexes (Me_2_SB(^R′^N,^3-R^I*)TiX_2_) supported on solid polymethylaluminoxane (sMAO) have been studied for ethylene homopolymerisation, H_2_ response, and ethylene/1-hexene copolymerisation in a high-throughput catalyst screening system.

The catalysts displayed very high ethylene homopolymerisation activities; maximum activity of 8400 kg_PE_ mol_Ti_^−1^ h^−1^ bar^−1^ for sMAO-Me_2_SB(^*t*Bu^N,I*)Ti(CH_2_Ph)_2_. sMAO-Me_2_SB(^*t*Bu^N,I*)TiCl_2_ displayed the best H_2_ response, displaying modest decreases in activity (6700 and 4900 kg_PE_ mol_Ti_^−1^ h^−1^ bar^−1^ with 0 and 1.6% H_2_ respectively), large decreases in polymer molecular weights (*M*_w_ of 2700 and 41 kDa with 0 and 1.6% H_2_ respectively), and narrow molecular weight distributions (*M*_w_/*M*_n_ of 2.4–3.2).

The addition of 1-hexene to the system caused a decrease in polymerisation activity and polymer molecular weights (activities of 6700 and 3600 kg_PE_ mol_Ti_^−1^ h^−1^ bar^−1^ and *M*_w_ of 2700 and 330 kDa for sMAO-Me_2_SB(^*t*Bu^N,I*)TiCl_2_ with 0 and 250 μL 1-hexene respectively), highlighting a negative comonomer effect.

The catalysts displayed high 1-hexene incorporation levels for supported systems with a maximum incorporation of 14.2 mol% for sMAO-Me_2_SB(^iPr^N,I*)TiCl_2_, demonstrating the formation of ethylene/1-hexene copolymers. A proportionate increase in 1-hexene incorporation with 1-hexene concentration was observed, demonstrating the potential capacity of these catalysts to controllably tune the amount of 1-hexene incorporated into the polymer chain to produce industrially relevant linear low-density polyethylene (LLDPE) materials.

## Conflicts of interest

There are no conflicts to declare.

## Supplementary Material

RA-011-D1RA00446H-s001
