# Supplementary material for: Synthesis of ultra-high molecular weight poly(ethylene)-co-(1-hexene) copolymers through high-throughput catalyst screening
Source: RSC Adv. 2021 Feb 2;11(10):5644–50. doi: 10.1039/d1ra00446h (PMC8694734; doi:10.1039/d1ra00446h)
Supplement: RA-011-D1RA00446H-s001 [file RA-011-D1RA00446H-s001.pdf]

Electronic Supplementary Material (ESI) for RSC Advances.
This journal is © The Royal Society of Chemistry 2021

Supplementary Information

Synthesis of ultra-high molecular weight poly(ethylene)-co-(1-hexene) copolymers through high-throughput catalyst screening

Thomas J. Williams,[a] Jessica V. Lamb,[a] Jean-Charles Buffet,[a] Tossapol Khamnaen,[b] and Dermot O'Hare[a] (*)

[a]*Chemistry Research Laboratory, Department of Chemistry, University of Oxford, 12 Mansfield Road, OX1 3TA Oxford, United Kingdom. Tel: +44(0) 1865 272686; E-mail: dermot.ohare@chem.ox.ac.uk*

[b]*SCG Chemicals Co., Ltd, 1 Siam Cement Rd, Bangkok 10800, Thailand*

Table of Contents

1. General experimental details S2
1.1 Air- and moisture-sensitive compounds S2
1.2 Reagents S2
1.3 $^{13}C\{^{1}H\}$ NMR spectroscopy S2
1.4 Gel-permeation chromatography (GPC) S3
1.5 Crystallisation elution fractionation (CEF) S2
1.6 High-throughput screening S2

2. Additional ethylene uptake rate profiles S5

3. Additional activity graphs S9

4. Molecular weights graphs S10

5. Crystallisation elution fractionation S16

6. High temperature NMR spectroscopy S22
7. Added exemplar characterisation S26
8. References S28

1. General experimental details

1.1 Air- and moisture-sensitive compounds

Air- and moisture-sensitive compounds were handled under an inert N_2 atmosphere, using standard Schlenk line techniques and an MBraun Unilab or triple MBraun LabMaster glovebox when required.

1.2 Reagents

^{Me2}SB(^{tBu}N,I*)TiCl$_2$ (**1**), ^{Me2}SB(^{tBu}N,I*)TiMe$_2$ (**2**), ^{Me2}SB(^{tBu}N,I*)Ti(CH$_2$Ph)$_2$ (**3**), ^{Me2}SB(^{tBu}N,I*)Ti(CH$_2$SiMe$_3$)$_2$ (**4**), ^{Me2}SB(^{tBu}N,I*)Ti(Cl)CH$_2$SiMe$_3$ (**5**), ^{Me2}SB(^{tBu}N,$^{3\text{-}Et}$I*)TiCl$_2$ (**6**), ^{Me2}SB(^{iPr}N,I*)TiCl$_2$ (**7**), and ^{Me2}SB(^{nBu}N,I*)TiCl$_2$ (**8**) were prepared according to literature procedures.[1, 2] Prior to use, solid polymethylaluminoxane (sMAO, SCG Chemicals Co., Ltd.) was dried under vacuum.

1.3 $^{13}C\{^1H\}$ NMR spectroscopy

^{13}C{^{1}H} NMR spectra were recorded with a 100 MHz Bruker Avance III 400 spectrometer equipped with a 5 mm high-temperature cryoprobe and a robotic sample changer with preheated carousel (24 positions). The samples (~30 mg) were dissolved at 120 °C in tetrachloroethane-1,2-d_2 (0.7 mL) added with 0.40 mg mL^{-1} BHT stabilizer and loaded in the carousel maintained at the same temperature. The spectra were taken sequentially with automated tuning, matching, and shimming. Operating conditions were as follows: 45° pulse; acquisition time, 2.7 s; relaxation delay, 5.0s; and 800 transients (corresponding to an experiment time of 60 minutes). Broad-band proton decoupling was achieved with a modified WALTZ16 sequence (BI_WALTZ16_32 by Bruker). Peak integration by full spectral simulation, and best-fit calculations of stereosequence distributions, were carried out using the SHAPE2004 and CONFSTAT (Ver. 3.1 for Windows) software packages, respectively. (Information on the software can be obtained from the Author: Prof. Michele Vacatello, Dipartimento di Chimica, Università di Napoli Federico II; e-mail: vacatello@chemistry.unina.it). Resonance assignment was based on the literature

1.4 Gel-permeation chromatography (GPC)

GPC curves were recorded with a Freeslate Rapid GPC setup, equipped with a set of two mixed-bed Agilent PLgel 10 μm columns and a Polymer Char IR4 detector. The upper deck of the setup features a sample dissolution station for up to 48 samples in 10 mL magnetically stirred glass vials. With robotic operation, preweighed polymer amounts (typically 1–4 mg) were dissolved in proper volumes of orthodichlorobenzene (ODCB) containing 0.40 mg mL^{-1} 4-methyl-2,6-di-*tert*-butyl-phenol (butylhydroxytoluene, BHT) stabiliser so as to obtain solutions at a concentration of 0.5–1.0 mg mL^{-1}. After 2 h at 150 °C under gentle stirring to ensure complete dissolution, the sample array was transferred to a thermostated bay at 145 °C, and the samples were sequentially injected into the column line at 145 °C and a flow rate of 1.0 mL min^{-1}. In post-trigger delay operation mode, the analysis time was 12.5 minutes per sample. Calibration was carried out with the universal method, using 10 monodisperse polystyrene samples (M_n between 1.3 and 3700 kDa).

1.5 Crystallisation elution fractionation (CEF)

CEF curves were collected with a Polymer Char CEF setup, equipped with a 42-well autosampler, an IR5 detector, and a dual-capillary viscometer detector. With robotic operation, preweighed polymer samples (typically 8–16 mg) were dissolved in ODCB added with 0.40 mg mL^{-1} BHT stabilizer so as to achieve a concentration of 2.0 mg mL^{-1}. After 90 minutes at 150 °C under vortexing in sealed vials to ensure complete dissolution, the samples were sequentially charged into the injection loop, where they were held at 95 °C for 5 minutes and then moved into the column. The crystallisation step entailed an 8.0 °C/min cooling ramp down to 35 °C at a flow rate of 0.24 mL min^{-1}; 1 min after reaching 35 °C, sample elution was started, with a 4 °C min^{-1} heating ramp up to 150 °C at a flow rate of 1.0 mL min^{-1}. The experiment time was 60 minutes per sample.

1.6 High-throughput screening

Polymerisation experiments were conducted at HTExplore (University of Naples, Italy) in a Freeslate Parallel Pressure Reactor (PPR) platform consisting of 48 reaction cells contained within a triple MBraun LabMaster Glovebox. The cells (geometric volume ~23 mL, working volume 5 mL) were individually controlled with monitoring of temperature and pressure. Slurry-phase polymerisations were run in semi-continuous mode. Two Vortex stir plates fitted with 6 × 8 racks

held the catalyst system components (pre-catalyst and scavenger). The injection system consisted of a dual-arm robot with specialised needles and injectors. Liquid solvents, diluents, and monomers were fed through syringe pumps, with gaseous monomers and diluents fed through direct lines with solenoid valves to individual cells.[3] Heptane solvent (5 mL) and TiBA scavenger (10 μmmol) were added to the PPR via robotic syringes, which were then heated to 80 °C and pressurized to 8.3 bar with ethylene. When required, H_2 was added to the reaction vessel via a 0.8 or 1.6% mixed H_2/N_2 feed. Pre-catalyst (0.075–0.40 mg in heptane slurry) and 1-hexene (when required) were injected with robotic syringes and chased by a certain amount of heptane solvent. The reactions were run for 1 hour or until a certain ethylene uptake was reached (8.3 bar for ethylene homopolymerisation and 5.5 bar for ethylene/1-hexene copolymerisation), following which the reaction was quenched with an excess of dry air. The polymer samples were transferred to a Genevac EZ-Plus centrifugal evaporator to remove volatiles and then dried under vacuum overnight.

2. Additional ethylene uptake rate profiles

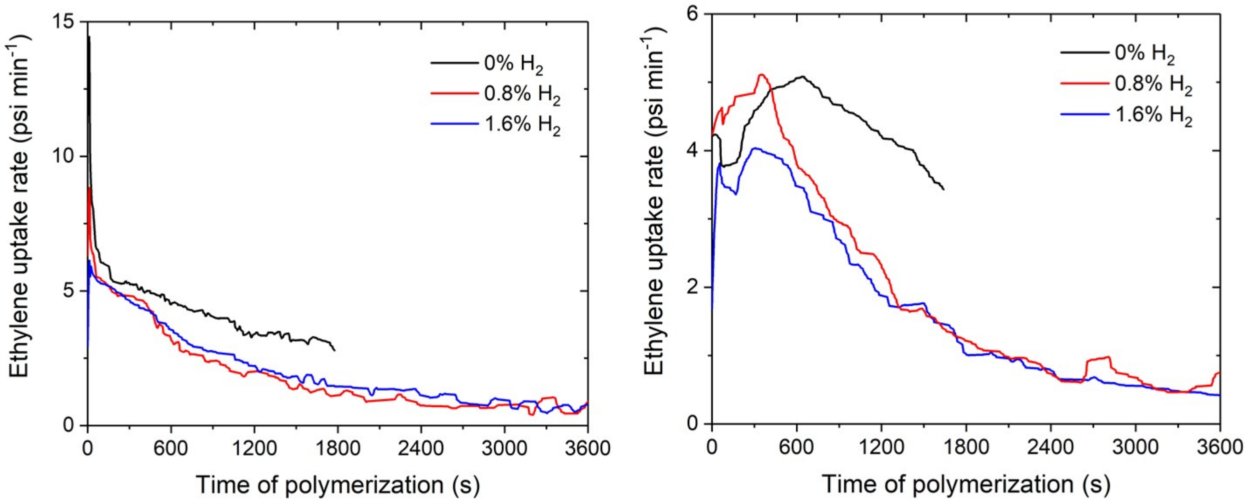

Fig. S1. Slurry-phase polymerisation ethylene uptake rate as a function of time of polymerisation using (left) sMAO-^{Me_2}SB($^{t\mathrm{Bu}}$N,I*)TiMe$_2$ ($\mathbf{2_{sMAO}}$) and (right) sMAO-^{Me_2}SB($^{t\mathrm{Bu}}$N,I*)Ti(CH$_2$Ph)$_2$ ($\mathbf{3_{sMAO}}$) with 0 (black), 0.8 (red), and 1.6% H_2 (blue). Polymerisation conditions: 8.3 bar ethylene, 0.075–0.40 mg pre-catalyst ([Al$_{sMAO}$]$_0$/[Ti]$_0$ = 200), 5.0 mL heptane, 10 μmol TiBA, and 80 °C. Reactions quenched at 8.3 bar ethylene uptake or after 60 minutes.

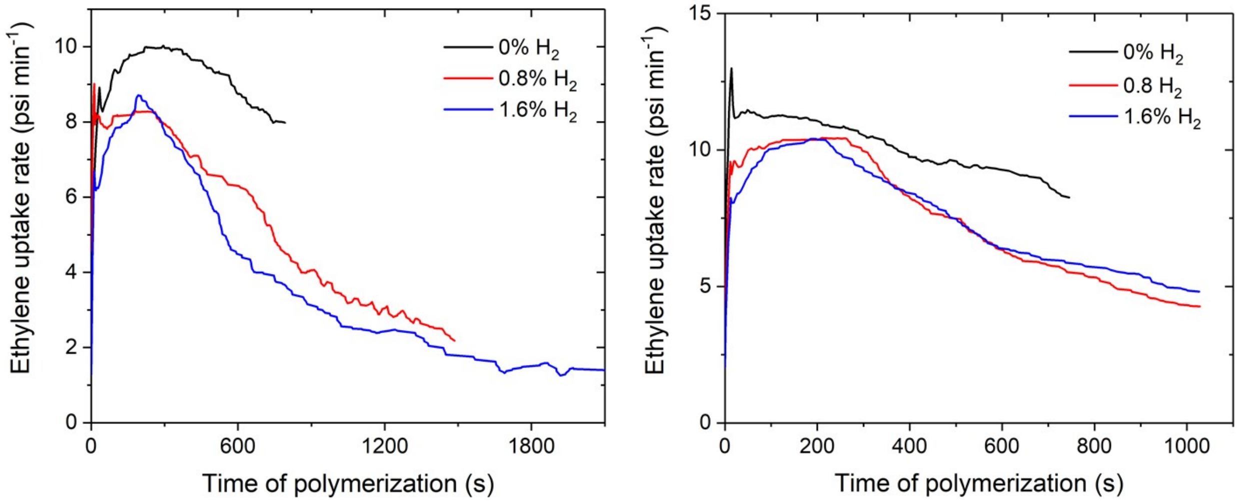

Fig. S2. Slurry-phase polymerisation ethylene uptake rate as a function of time of polymerisation using (left) sMAO-^{Me_2}SB(^{tBu}N,I*)Ti(CH_2SiMe_3)$_2$ (**4**$_{\mathbf{sMAO}}$) and (right) sMAO-^{Me_2}SB(^{tBu}N,I*)Ti(Cl)CH_2SiMe_3 (**5**$_{\mathbf{sMAO}}$) with 0 (black), 0.8 (red), and 1.6% H_2 (blue). Polymerisation conditions: 8.3 bar ethylene, 0.30 mg pre-catalyst ([Al$_{sMAO}$]$_0$/[Ti]$_0$ = 200), 5.0 mL heptane, 10 µmol TiBA, and 80 °C. Reactions quenched at 8.3 bar ethylene uptake or after 60 minutes.

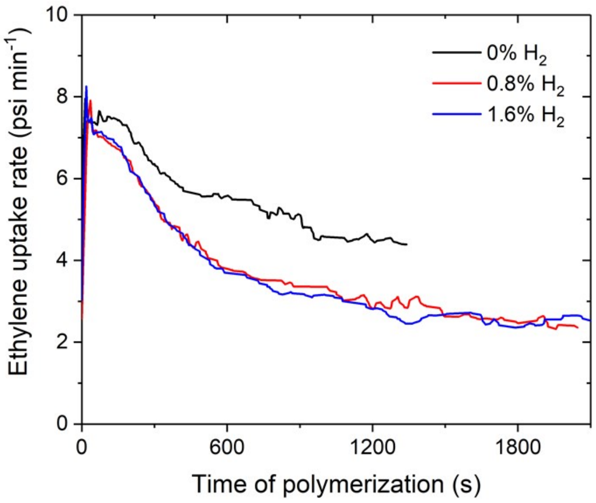

Fig. S3. Slurry-phase polymerisation ethylene uptake rate as a function of time of polymerisation using sMAO-^{Me_2}SB(^{tBu}N,$^{3\text{-}Et}$I*)$TiCl_2$ (**6**$_{\mathbf{sMAO}}$) with 0 (black), 0.8 (red), and 1.6% H_2 (blue). Polymerisation conditions: 8.3 bar ethylene, 0.30 mg pre-catalyst ([Al$_{sMAO}$]$_0$/[Ti]$_0$ = 200), 5.0 mL heptane, 10 µmol TiBA, and 80 °C. Reactions quenched at 8.3 bar ethylene uptake or after 60 minutes.

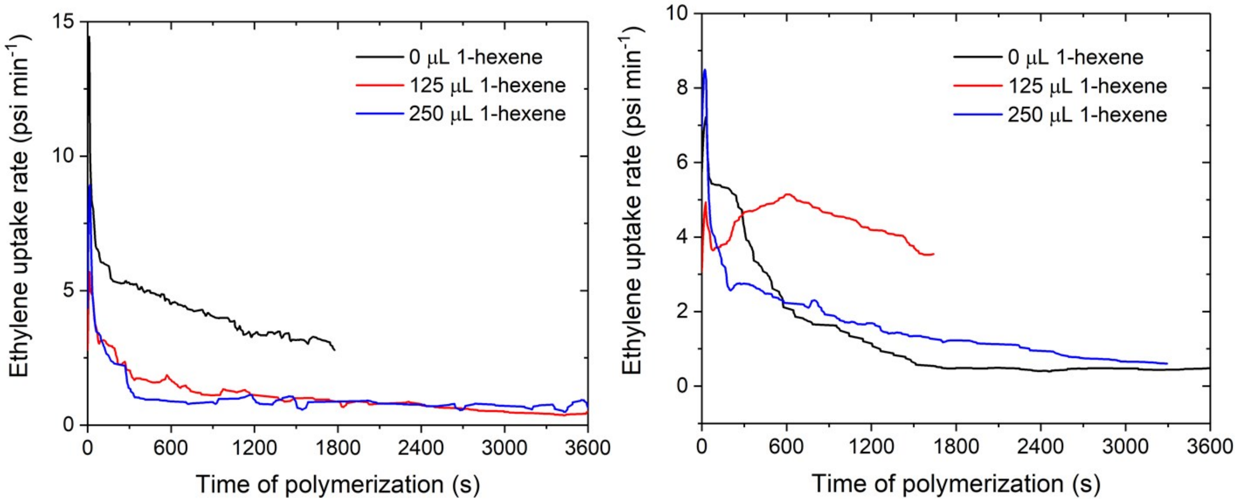

Fig. S4. Slurry-phase polymerisation ethylene uptake rate as a function of time of polymerisation using (left) sMAO-^{Me_2}SB(^{tBu}N,I*)TiMe$_2$ (**2**$_{\mathbf{sMAO}}$) and (right) sMAO-^{Me_2}SB(^{tBu}N,I*)Ti(CH_2Ph)$_2$ (**3**$_{\mathbf{sMAO}}$) with 0 (black), 125 (red), and 250 μL 1-hexene (blue). Polymerisation conditions: 8.3 bar ethylene, 0.075–0.40 mg pre-catalyst ([Al$_{sMAO}$]$_0$/[Ti]$_0$ = 200), 5.0 mL heptane, 10 μmol TiBA, and 80 °C. Reactions quenched at 5.5 bar ethylene uptake or after 60 minutes.

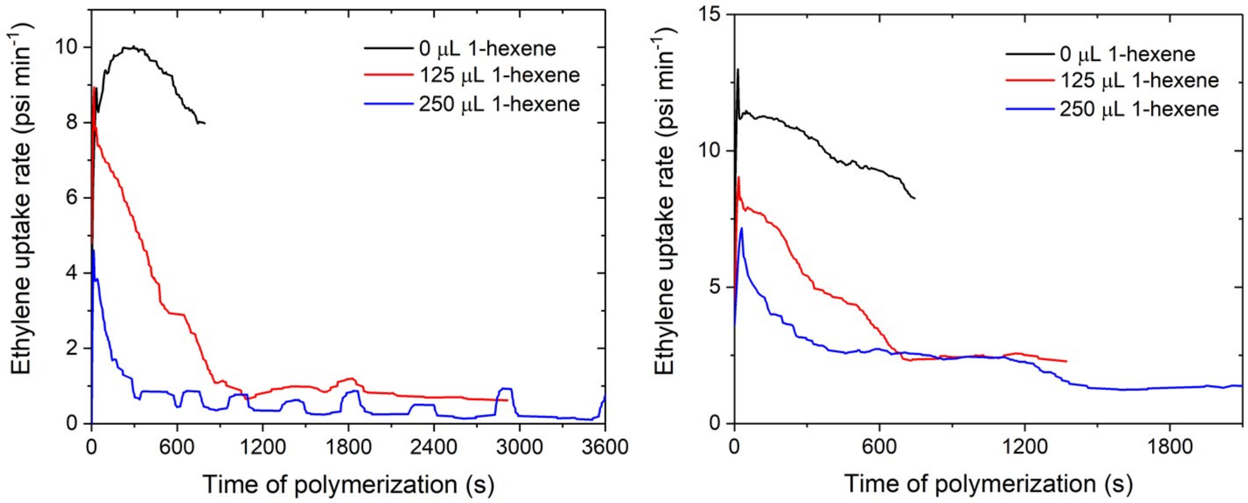

Fig. S5. Slurry-phase polymerisation ethylene uptake rate as a function of time of polymerisation using (left) sMAO-^{Me_2}SB(^{tBu}N,I*)Ti(CH_2SiMe_3)$_2$ (**4**$_{\mathbf{sMAO}}$) and (right) sMAO-^{Me_2}SB(^{tBu}N,I*)Ti(Cl)CH_2SiMe_3 (**5**$_{\mathbf{sMAO}}$) with 0 (black), 125 (red), and 250 μL 1-hexene (blue). Polymerisation conditions: 8.3 bar ethylene, 0.30 mg pre-catalyst ([Al$_{sMAO}$]$_0$/[Ti]$_0$ = 200), 5.0 mL heptane, 10 μmol TiBA, and 80 °C. Reactions quenched at 5.5 bar ethylene uptake or after 60 minutes.

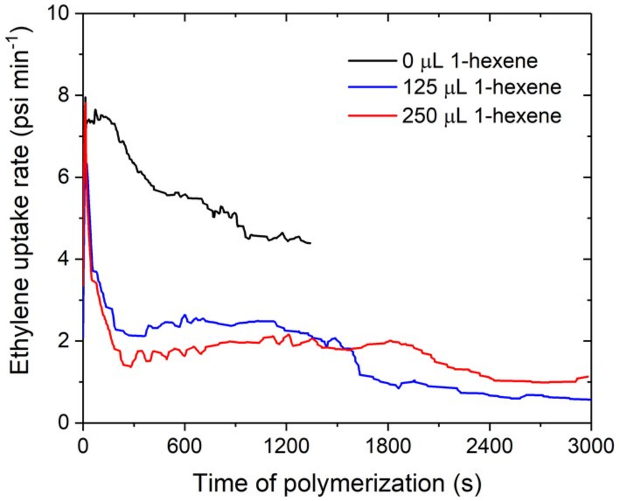

Fig. S6. Slurry-phase polymerisation ethylene uptake rate as a function of time of polymerisation using sMAO-^{Me_2}SB(^{tBu}N,$^{3\text{-}Et}$I*)$TiCl_2$ (**6$_{sMAO}$**) with 0 (black), 125 (red), and 250 μL 1-hexene (blue). Polymerisation conditions: 8.3 bar ethylene, 0.30 mg pre-catalyst ($[Al_{sMAO}]_0/[Ti]_0 = 200$), 5.0 mL heptane, 10 μmol TiBA, and 80 °C. Reactions quenched at 5.5 bar ethylene uptake or after 60 minutes.

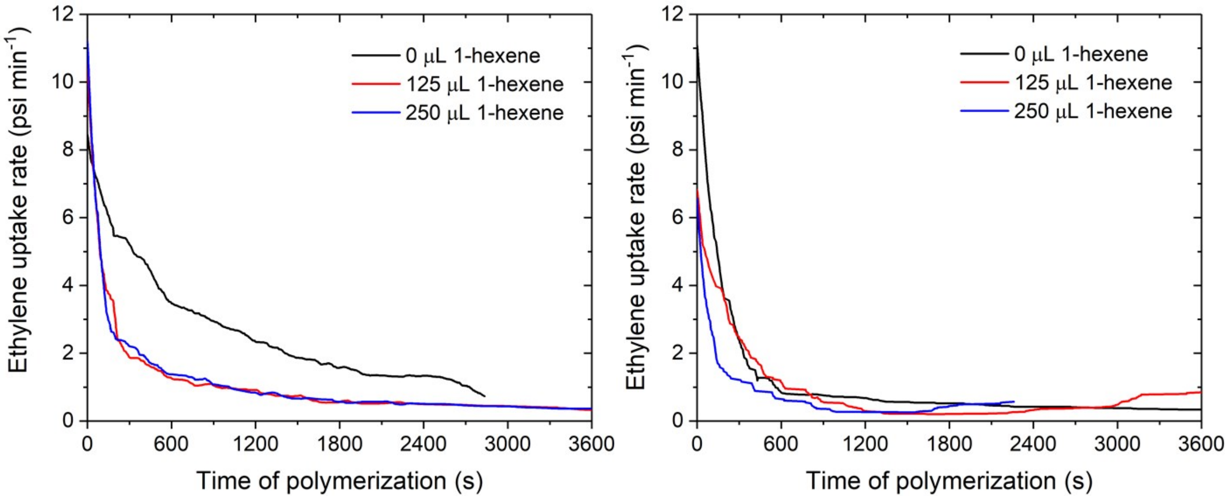

Fig. S7. Slurry-phase polymerisation ethylene uptake rate as a function of time of polymerisation using (left) sMAO-^{Me_2}SB(^{iPr}N,I*)$TiCl_2$ (**7$_{sMAO}$**) and (right) sMAO-^{Me_2}SB(^{nBu}N,I*)$TiCl_2$ (**8$_{sMAO}$**) with 0 (black), 125 (red), and 250 μL 1-hexene (blue). Polymerisation conditions: 8.3 bar ethylene, 0.20–0.40 mg pre-catalyst ($[Al_{sMAO}]_0/[Ti]_0 = 200$), 5.0 mL heptane, 10 μmol TiBA, and 80 °C. Reactions quenched at 5.5 bar ethylene uptake or after 60 minutes.

3. Additional activity graphs

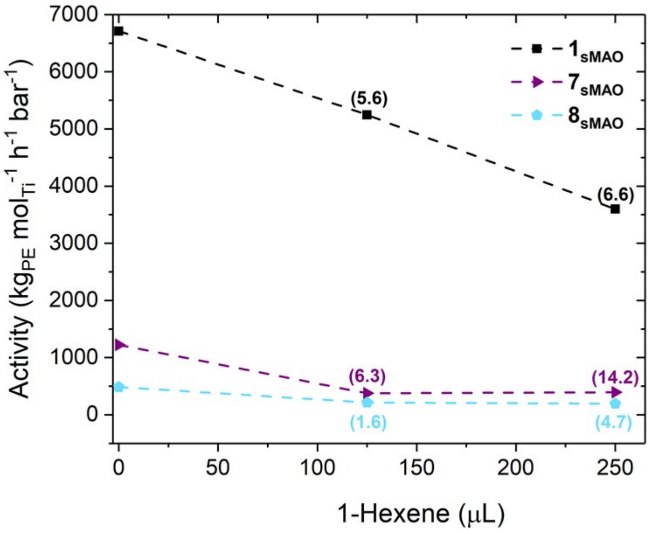

Fig. S8. Slurry-phase ethylene polymerisation activity as a function of 1-hexene (μL) using sMAO supported ^{Me_2}SB(^{tBu}N,I*)TiCl$_2$ (**1$_{sMAO}$**) (black square), ^{Me_2}SB(^{iPr}N,I*)TiCl$_2$ (**7$_{sMAO}$**) (purple right triangle), and ^{Me_2}SB(^{nBu}N,I*)TiCl$_2$ (**8$_{sMAO}$**) (teal pentagon). 1-hexene incorporation (mol%) shown in parenthesis. Polymerisation conditions: 8.3 bar ethylene, 0.075–0.40 mg pre-catalyst ($[Al_{sMAO}]_0/[Ti]_0 = 200$), 5.0 mL heptane, 10 μmol TiBA, and 80 °C. Reactions quenched at 5.5 bar ethylene uptake or after 60 minutes.

4. Molecular weights graphs

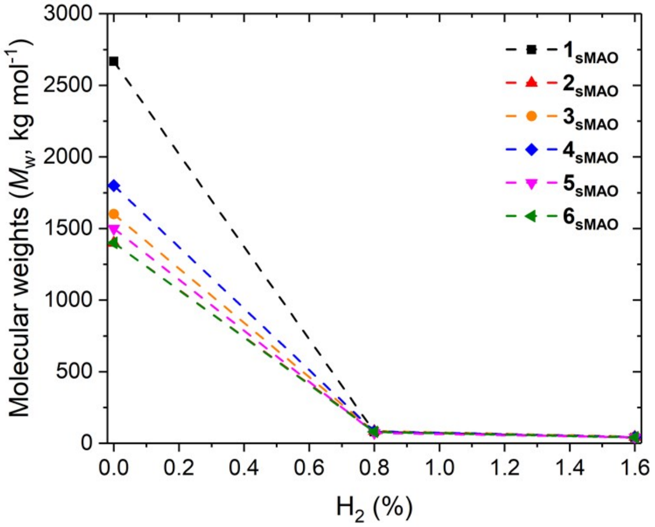

Fig. S9. Polymer molecular weights (M_w) as a function of H_2 (%) using sMAO supported ^{Me_2}SB(^{tBu}N,I*)TiCl$_2$ (**1**$_{\mathbf{sMAO}}$) (black square), ^{Me_2}SB(^{tBu}N,I*)TiMe$_2$ (**2**$_{\mathbf{sMAO}}$) (red up triangle), ^{Me_2}SB(^{tBu}N,I*)Ti(CH$_2$Ph)$_2$ (**3**$_{\mathbf{sMAO}}$) (orange circle), ^{Me_2}SB(^{tBu}N,I*)Ti(CH$_2$SiMe$_3$)$_2$ (**4**$_{\mathbf{sMAO}}$) (blue diamond), ^{Me_2}SB(^{tBu}N,I*)Ti(Cl)CH$_2$SiMe$_3$ (**5**$_{\mathbf{sMAO}}$) (pink down triangle), and ^{Me_2}SB(^{tBu}N,$^{3\text{-}Et}$I*)TiCl$_2$ (**6**$_{\mathbf{sMAO}}$) (green left triangle). Polymerisation conditions: 8.3 bar ethylene, 0.075–0.40 mg pre-catalyst ([Al$_{sMAO}$]$_0$/[Ti]$_0$ = 200), 5.0 mL heptane, 10 µmol TiBA, and 80 °C. Reactions quenched at 8.3 bar ethylene uptake or after 60 minutes.

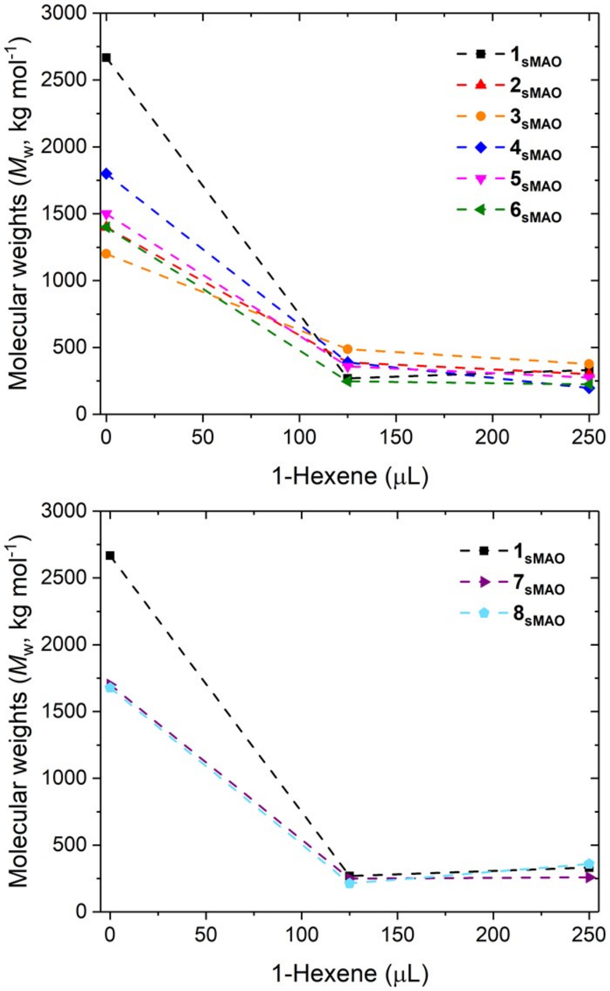

Fig. S10. Polymer molecular weights (M_w) as a function of 1-hexene (μL) using sMAO supported (left) ^{Me_2}SB($^{t\mathrm{Bu}}$N,I*)TiCl$_2$ ($\mathbf{1_{sMAO}}$) (black square), ^{Me_2}SB($^{t\mathrm{Bu}}$N,I*)TiMe$_2$ ($\mathbf{2_{sMAO}}$) (red up triangle), ^{Me_2}SB($^{t\mathrm{Bu}}$N,I*)Ti(CH$_2$Ph)$_2$ ($\mathbf{3_{sMAO}}$) (orange circle), ^{Me_2}SB($^{t\mathrm{Bu}}$N,I*)Ti(CH$_2$SiMe$_3$)$_2$ ($\mathbf{4_{sMAO}}$) (blue diamond), ^{Me_2}SB($^{t\mathrm{Bu}}$N,I*)Ti(Cl)CH$_2$SiMe$_3$ ($\mathbf{5_{sMAO}}$) (pink down triangle), and ^{Me_2}SB($^{t\mathrm{Bu}}$N,$^{3\text{-}\mathrm{Et}}$I*)TiCl$_2$ ($\mathbf{6_{sMAO}}$) (green left triangle) and (right) ^{Me_2}SB($^{t\mathrm{Bu}}$N,I*)TiCl$_2$ ($\mathbf{1_{sMAO}}$) (black square), ^{Me_2}SB($^{i\mathrm{Pr}}$N,I*)TiCl$_2$ ($\mathbf{7_{sMAO}}$) (purple right triangle) and ^{Me_2}SB($^{n\mathrm{Bu}}$N,I*)TiCl$_2$ ($\mathbf{8_{sMAO}}$) (teal pentagon). Polymerisation conditions: 8.3 bar ethylene, 0.075–0.40 mg pre-catalyst ([Al$_{sMAO}$]$_0$/[Ti]$_0$ = 200), 5.0 mL heptane, 10 μmol TiBA, and 80 °C. Reactions quenched at 5.5 bar ethylene uptake or after 60 minutes.

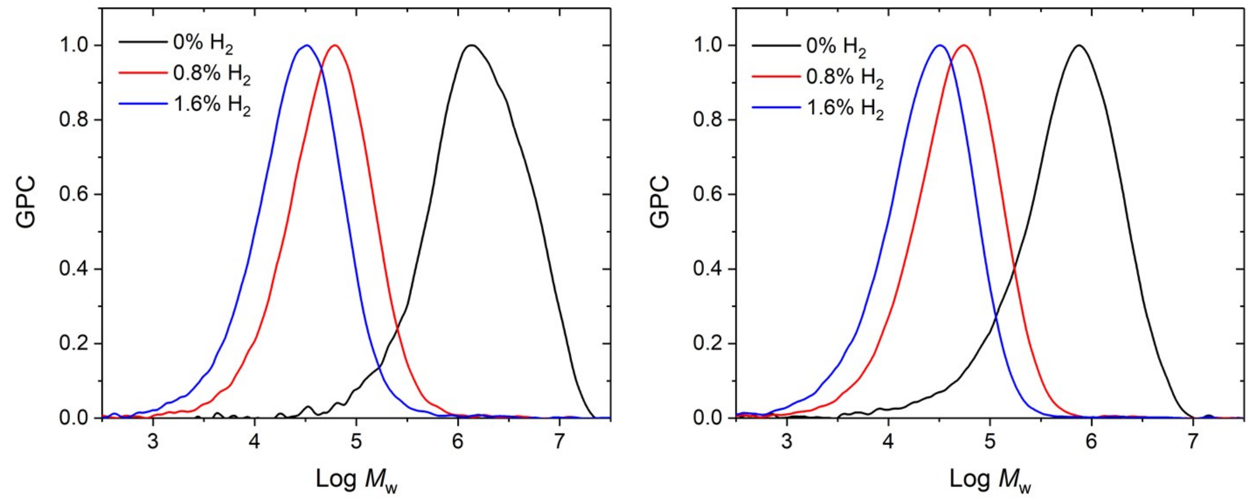

Fig. S11. Molecular weights distribution for the polymers produced using sMAO supported (left) $^{Me_2}SB(^{tBu}N,I^*)TiCl_2$ ($\mathbf{1_{sMAO}}$) and (right) $^{Me_2}SB(^{tBu}N,I^*)TiMe_2$ ($\mathbf{2_{sMAO}}$) with 0 (black), 0.8, (red) and 1.6% H_2 (blue). Normalized for clarity. Polymerisation conditions: 8.3 bar ethylene, 0.20–0.40 mg pre-catalyst ($[Al_{sMAO}]_0/[Ti]_0$ = 200), 5.0 mL heptane, 10 µmol TiBA, and 80 °C. Reactions quenched at 8.3 bar ethylene uptake or after 60 minutes.

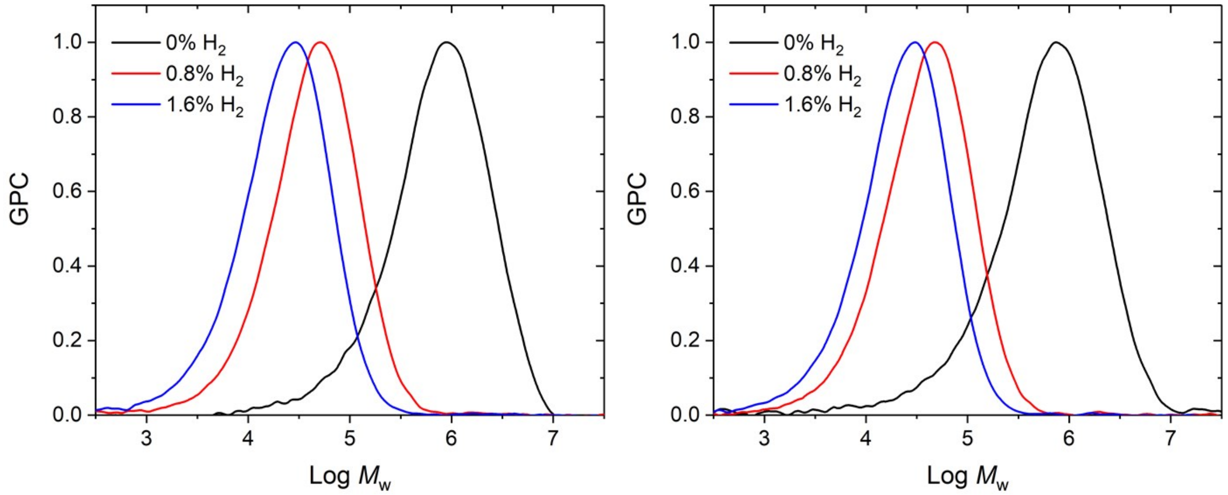

Fig. S12. Molecular weights distribution for the polymers produced using sMAO supported (left) $^{Me_2}SB(^{tBu}N,I^*)Ti(CH_2SiMe_3)_2$ (**4_{sMAO}**) and (right) $^{Me_2}SB(^{tBu}N,I^*)TiCl(CH_2SiMe_3)$ (**5_{sMAO}**) with 0 (black), 0.8 (red), and 1.6% H_2 (blue). Normalized for clarity. Polymerisation conditions: 8.3 bar ethylene, 0.30 mg pre-catalyst ($[Al_{sMAO}]_0/[Ti]_0 = 200$), 5.0 mL heptane, 10 μmol TiBA, and 80 °C. Reactions quenched at 8.3 bar ethylene uptake or after 60 minutes.

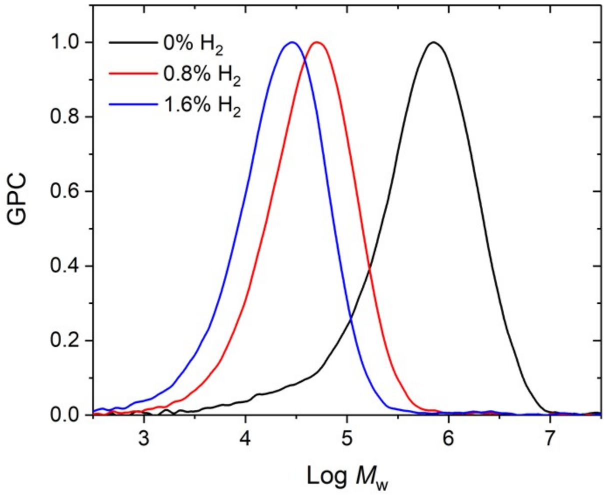

Fig. S13. Molecular weights distribution for the polymers produced using sMAO supported $^{Me_2}SB(^{tBu}N,^{3\text{-}Et}I^*)TiCl_2$ (**6_{sMAO}**) with 0 (black), 0.8 (red), and 1.6% H_2 (blue). Normalized for clarity. Polymerisation conditions: 8.3 bar ethylene, 0.30 mg pre-catalyst ($[Al_{sMAO}]_0/[Ti]_0 = 200$), 5.0 mL heptane, 10 μmol TiBA, and 80 °C. Reactions quenched at 8.3 bar ethylene uptake or after 60 minutes.

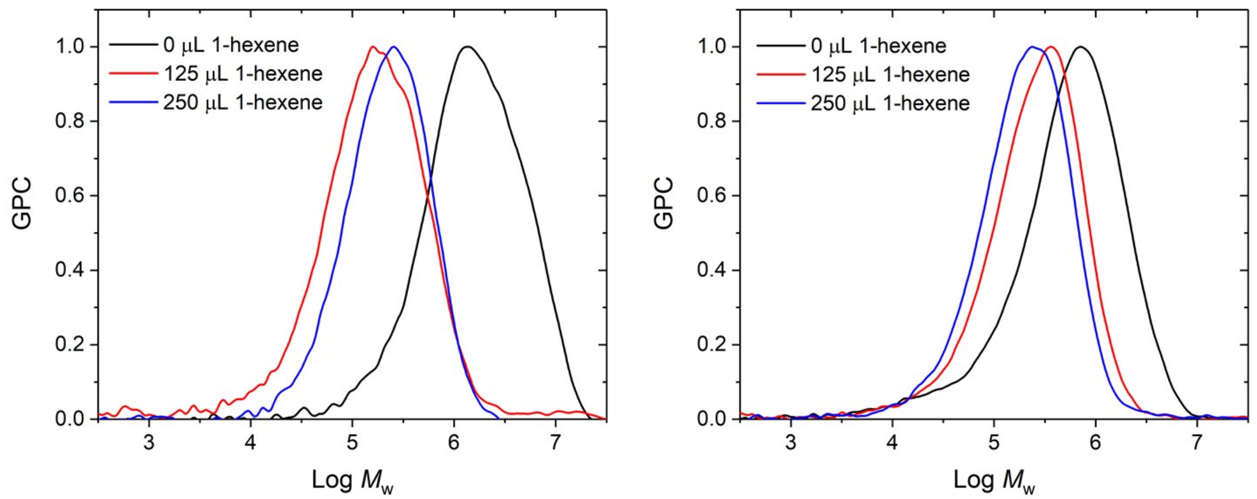

Fig. S14. Molecular weights distribution for the polymers produced using sMAO supported (left) ${}^{Me_2}SB({}^{tBu}N,I^*)TiCl_2$ ($\mathbf{1_{sMAO}}$) and (right) ${}^{Me_2}SB({}^{tBu}N,I^*)TiMe_2$ ($\mathbf{2_{sMAO}}$) with 0 (black), 125 (red), and 250 μL 1-hexene (blue). Normalized for clarity. Polymerisation conditions: 8.3 bar ethylene, 0.20–0.40 mg pre-catalyst ($[Al_{sMAO}]_0/[Ti]_0$ = 200), 5.0 mL heptane, 10 μmol TiBA, and 80 °C. Reactions quenched at 5.5 bar ethylene uptake or after 60 minutes.

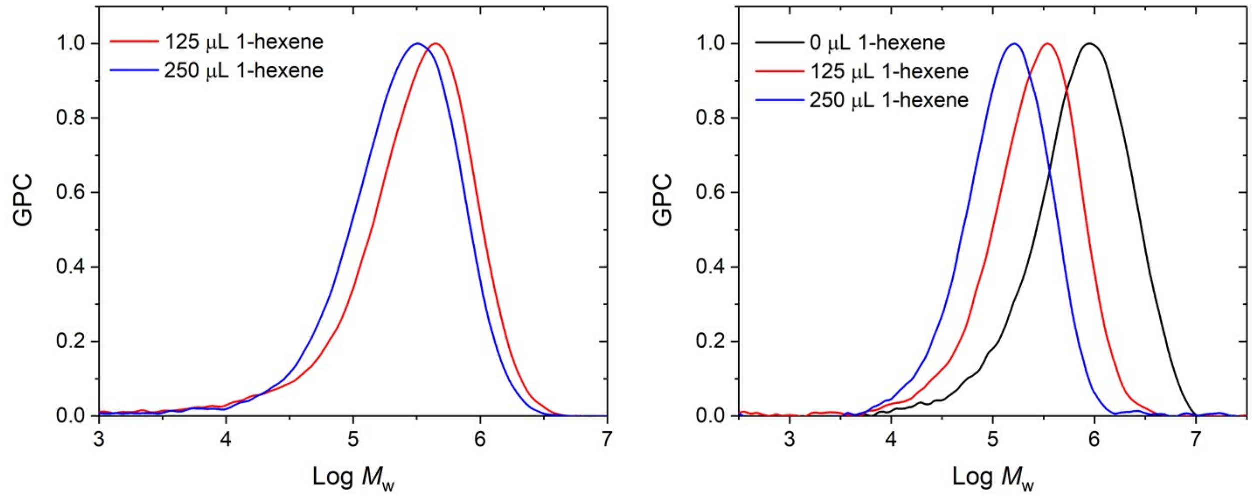

Fig. S15. Molecular weights distribution for the polymers produced using sMAO supported (left) ${}^{Me_2}SB({}^{tBu}N,I^*)Ti(CH_2Ph)_2$ ($\mathbf{3_{sMAO}}$) and (right) ${}^{Me_2}SB({}^{tBu}N,I^*)Ti(CH_2SiMe_3)_2$ ($\mathbf{4_{sMAO}}$) with 0 (black), 125 (red), and 250 μL 1-hexene (blue). Normalized for clarity. Polymerisation conditions: 8.3 bar ethylene, 0.075–0.30 mg pre-catalyst ($[Al_{sMAO}]_0/[Ti]_0$ = 200), 5.0 mL heptane, 10 μmol TiBA, and 80 °C. Reactions quenched at 5.5 bar ethylene uptake or after 60 minutes.

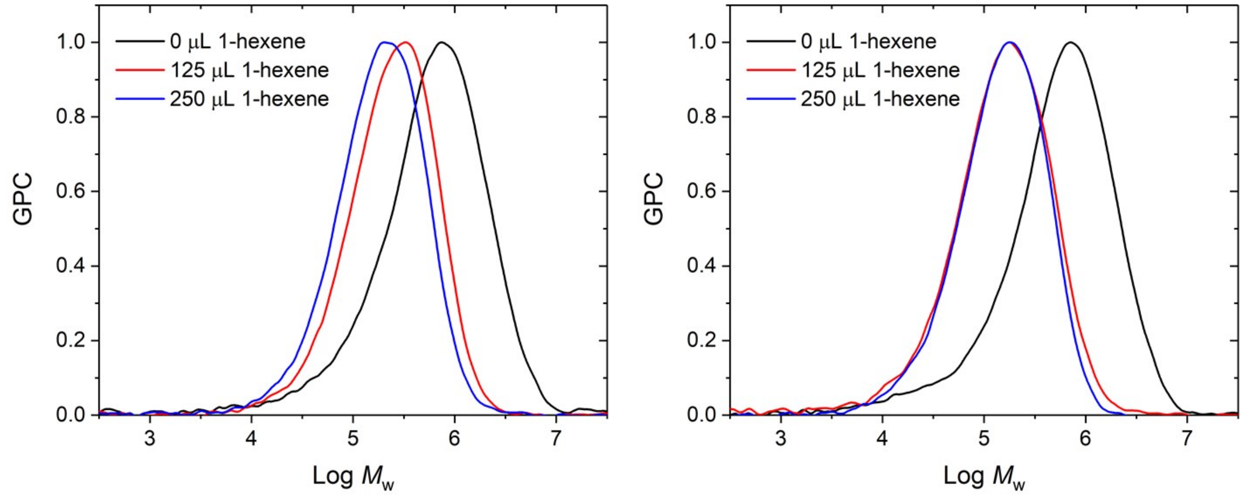

Fig. S16. Molecular weights distribution for the polymers produced using sMAO supported (left) $^{Me_2}SB(^{tBu}N,I^*)TiCl(CH_2SiMe_3)$ (**5_{sMAO}**) and (right) $^{Me_2}SB(^{tBu}N,^{3\text{-}Et}I^*)TiCl_2$ (**6_{sMAO}**) with 0 (black), 125 (red), and 250 μL 1-hexene (blue). Normalized for clarity. Polymerisation conditions: 8.3 bar ethylene, 0.30 mg pre-catalyst ($[Al_{sMAO}]_0/[Ti]_0 = 200$), 5.0 mL heptane, 10 μmol TiBA, and 80 °C. Reactions quenched at 5.5 bar ethylene uptake or after 60 minutes.

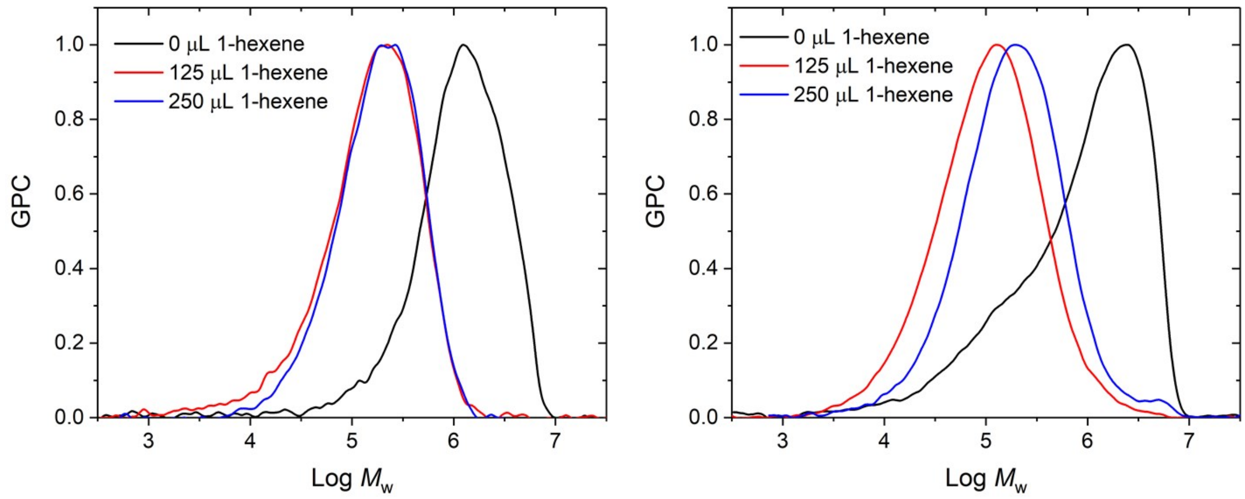

Fig. S17. Molecular weights distribution for the polymers produced using sMAO supported (left) $^{Me_2}SB(^{iPr}N,I^*)TiCl_2$ (**7_{sMAO}**) and (right) $^{Me_2}SB(^{nBu}N,I^*)TiCl_2$ (**8_{sMAO}**) with 0 (black), 125 (red), and 250 μL 1-hexene (blue). Normalized for clarity. Polymerisation conditions: 8.3 bar ethylene, 0.40 mg pre-catalyst ($[Al_{sMAO}]_0/[Ti]_0 = 200$), 5.0 mL heptane, 10 μmol TiBA, and 80 °C. Reactions quenched at 5.5 bar ethylene uptake or after 60 minutes.

5. Crystallisation elution fraction

Table S1. Temperature at maximum elution ($T_{el,max}$) and amorphous fraction (AF) for the polymers produced using sMAO supported complexes with H_2 variation.

Catalyst	H_2 (%)	$T_{el,max}$ (°C)	AF (wt%)
1_{sMAO}	0	112.1	0.9
	0.8	111.3	0.5
	1.6	110.9	1.2
2_{sMAO}	0	113.3	0.2
	0.8	112.1	0.5
	1.6	111.8	0.7
3_{sMAO}	0	111.4	0.1
	0.8	110.9	0.4
	1.6	110.5	1.1
4_{sMAO}	0	113.3	0.2
	0.8	112.2	0.4
	1.6	111.7	0.8
5_{sMAO}	0	113.3	0.2

	0.8	112.3	0.4
	1.6	111.9	0.6
**6_{sMAO}**	0	113.4	0.2
	0.8	112.1	0.4
	1.6	111.8	0.7

Polymerisation conditions: 8.3 bar ethylene, 0.075−0.40 mg pre-catalyst ($[Al_{sMAO}]_0/[Ti]_0 = 200$), 5.0 mL heptane, 10 μmol TiBA, and 80 °C. Reactions quenched at 8.3 bar ethylene uptake or after 60 minutes.

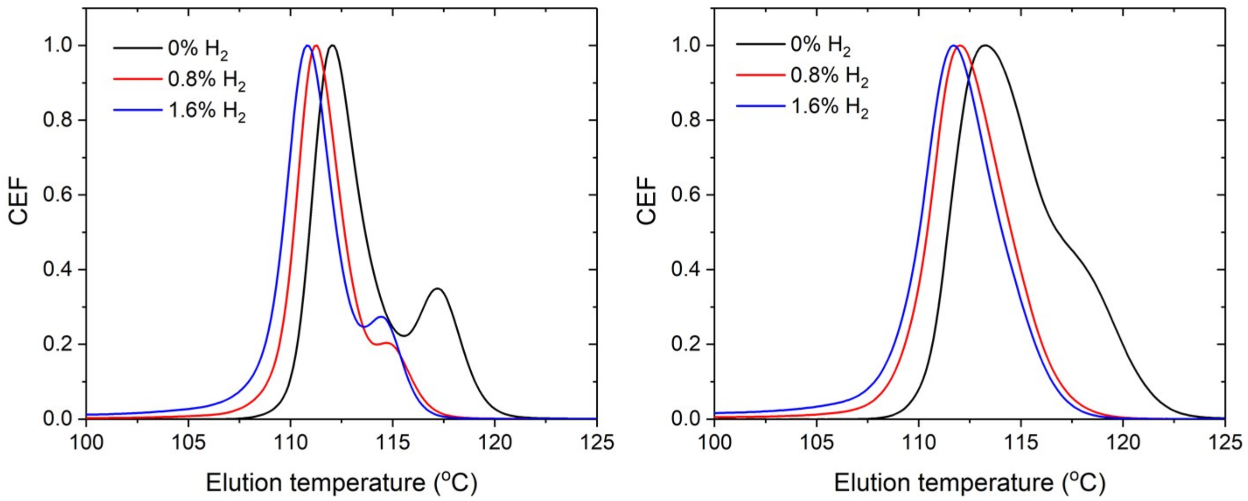

Fig. S18. Crystallisation elution fractionation plot for the polymers produced using sMAO supported (left) $^{Me_2}SB(^{tBu}N,I^*)TiCl_2$ (**1_{sMAO}**) and (right) $^{Me_2}SB(^{tBu}N,I^*)TiMe_2$ (**2_{sMAO}**) with 0 (black), 0.8 (red), and 1.6% H_2 (blue). Normalized for clarity. Polymerisation conditions: 8.3 bar ethylene, 0.20–0.40 mg pre-catalyst ($[Al_{sMAO}]_0/[Ti]_0 = 200$), 5.0 mL heptane, 10 μmol TiBA, and 80 °C. Reactions quenched at 8.3 bar ethylene uptake or after 60 minutes.

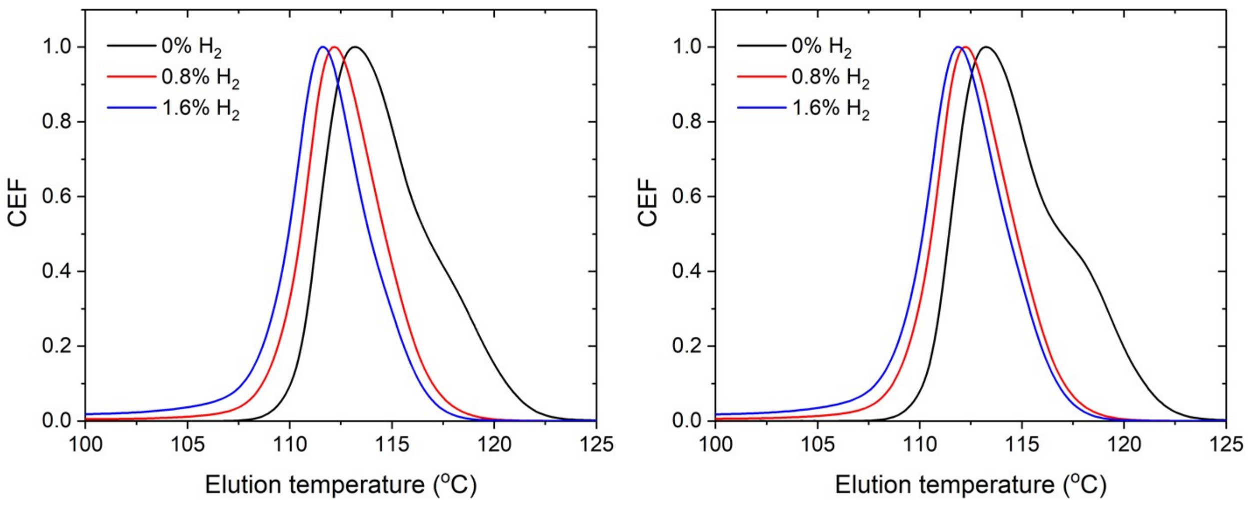

Fig. S19. Crystallisation elution fractionation plot for the polymers produced using sMAO supported (left) ^{Me_2}SB(^{tBu}N,I*)Ti(CH_2SiMe_3)$_2$ (**4**$_{\mathbf{sMAO}}$) and (right) ^{Me_2}SB(^{tBu}N,I*)TiCl(CH_2SiMe_3) (**5**$_{\mathbf{sMAO}}$) with 0 (black), 0.8 (red), and 1.6% H_2 (blue). Normalized for clarity. Polymerisation conditions: 8.3 bar ethylene, 0.30 mg pre-catalyst ($[Al_{sMAO}]_0/[Ti]_0$ = 200), 5.0 mL heptane, 10 µmol TiBA, and 80 °C. Reactions quenched at 8.3 bar ethylene uptake or after 60 minutes.

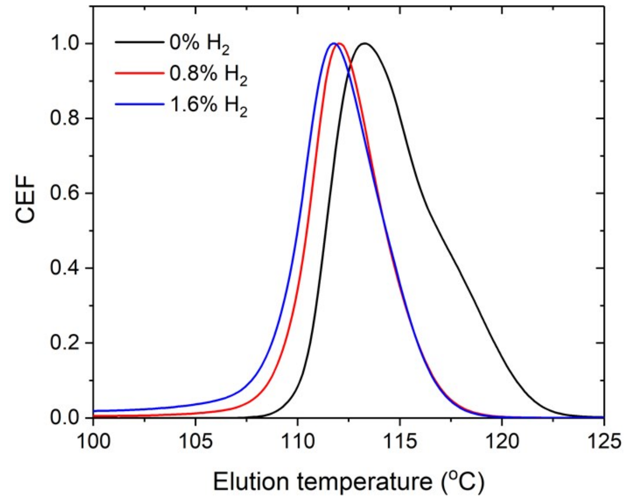

Fig. S20. Crystallisation elution fractionation plot for the polymers produced using sMAO supported ^{Me_2}SB(^{tBu}N,$^{3\text{-}Et}$I*)TiCl_2 (**6**$_{\mathbf{sMAO}}$) with 0 (black), 0.8 (red) and 1.6% H_2 (blue). Normalized for clarity. Polymerisation conditions: 8.3 bar ethylene, 0.30 mg pre-catalyst ($[Al_{sMAO}]_0/[Ti]_0$ = 200), 5.0 mL heptane, 10 µmol TiBA and 80 °C. Reactions quenched at 8.3 bar ethylene uptake or after 60 minutes.

Table S2. Amorphous fraction (AF) for the polymers produced using sMAO supported complexes with 1-hexene variation.

Catalyst	1-Hexene (µL)	AF (wt%)
$\mathbf{1}_{\mathbf{sMAO}}$	0	0.9
	125	21.2
	250	19.8
$\mathbf{2}_{\mathbf{sMAO}}$	0	0.2
	125	0.6
	250	10.0
$\mathbf{3}_{\mathbf{sMAO}}$	0	0.1
	125	0.3
	250	25.3
$\mathbf{4}_{\mathbf{sMAO}}$	0	0.2
	125	0.7
	250	27.2
$\mathbf{5}_{\mathbf{sMAO}}$	0	0.2
	125	0.9
	250	10.7
$\mathbf{6}_{\mathbf{sMAO}}$	0	0.2

	125	3.2
	250	56.1
**7_{sMAO}**	0	0.2
	125	55.2
	250	94.8

Polymerisation conditions: 8.3 bar ethylene, 0.075−0.40 mg pre-catalyst ($[Al_{sMAO}]_0/[Ti]_0 = 200$), 5.0 mL heptane, 10 μmol TiBA, and 80 °C. Reactions quenched at 5.5 bar ethylene uptake or after 60 minutes.

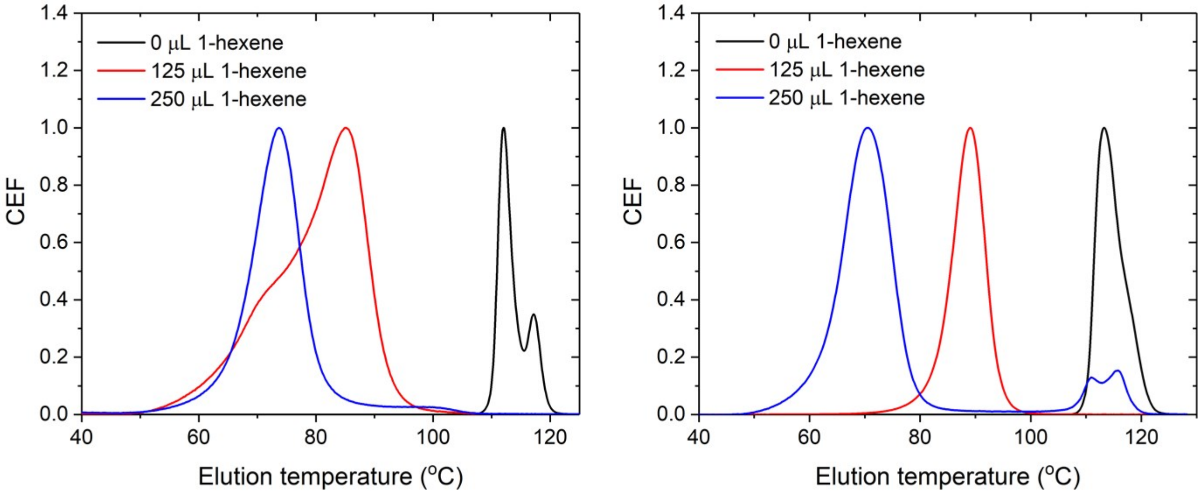

Fig. S21. Crystallisation elution fractionation plot for the polymers produced using sMAO supported (left) ^{Me_2}SB(^{tBu}N,I*)TiCl$_2$ (**1_{sMAO}**) and (right) ^{Me_2}SB(^{tBu}N,I*)TiMe$_2$ (**2_{sMAO}**) with 0 (black), 125 (red), and 250 μL 1-hexene (blue). Normalized for clarity. Polymerisation conditions: 8.3 bar ethylene, 0.20–0.40 mg pre-catalyst ($[Al_{sMAO}]_0/[Ti]_0 = 200$), 5.0 mL heptane, 10 μmol TiBA, and 80 °C. Reactions quenched at 5.5 bar ethylene uptake or after 60 minutes.

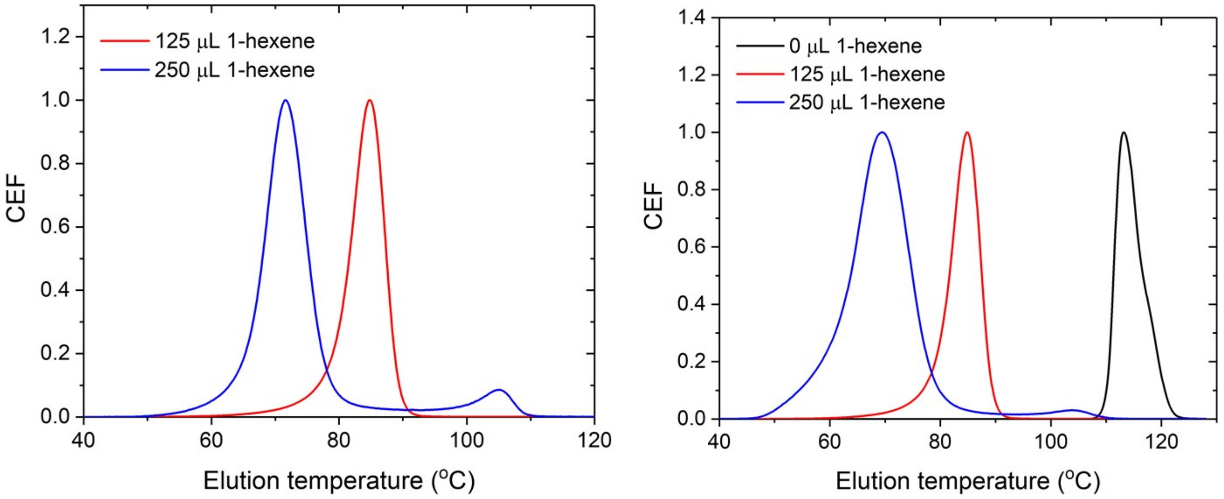

Fig. S22. Crystallisation elution fractionation plot for the polymers produced using sMAO supported (left) ^{Me_2}SB(^{tBu}N,I*)Ti(CH_2Ph)$_2$ (**3**$_{\mathbf{sMAO}}$) and (right) ^{Me_2}SB(^{tBu}N,I*)Ti(CH_2SiMe_3)$_2$ (**4**$_{\mathbf{sMAO}}$) with 0 (black), 125 (red), and 250 μL 1-hexene (blue). Normalized for clarity. Polymerisation conditions: 8.3 bar ethylene, 0.075–0.30 mg pre-catalyst ([Al_{sMAO}]$_0$/[Ti]$_0$ = 200), 5.0 mL heptane, 10 μmol TiBA, and 80 °C. Reactions quenched at 5.5 bar ethylene uptake or after 60 minutes.

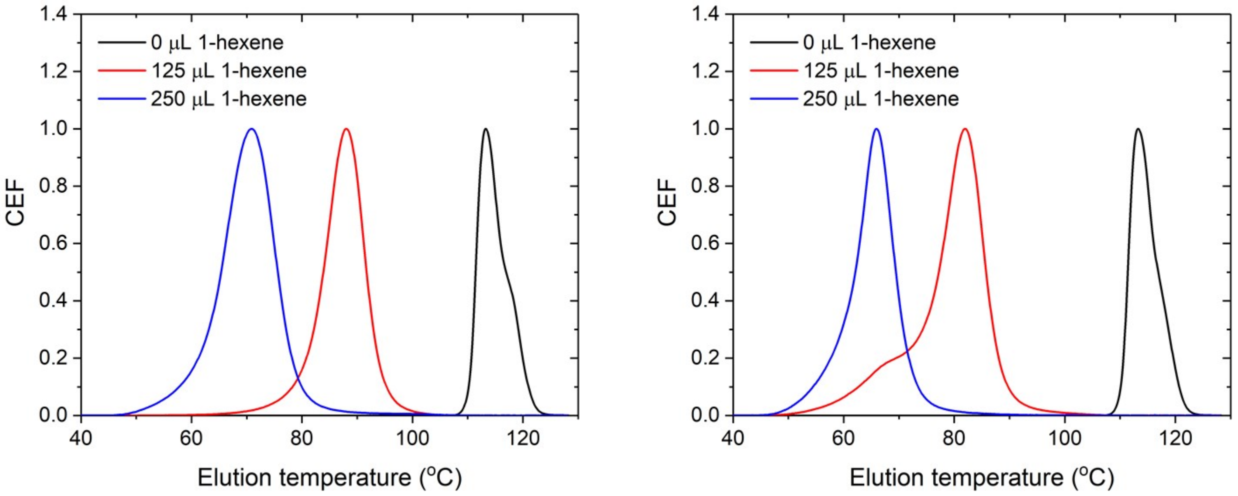

Fig. S23. Crystallisation elution fractionation plot for the polymers produced using sMAO supported (left) ^{Me_2}SB(^{tBu}N,I*)TiCl(CH_2SiMe_3) (**5**$_{\mathbf{sMAO}}$) and (right) ^{Me_2}SB(^{tBu}N,${}^{3\text{-}Et}$I*)TiCl$_2$ (**6**$_{\mathbf{sMAO}}$) with 0 (black), 125 (red), and 250 μL 1-hexene (blue). Normalized for clarity. Polymerisation conditions: 8.3 bar ethylene, 0.30 mg pre-catalyst ([Al_{sMAO}]$_0$/[Ti]$_0$ = 200), 5.0 mL heptane, 10 μmol TiBA, and 80 °C. Reactions quenched at 5.5 bar ethylene uptake or after 60 minutes.

6. High temperature NMR spectroscopy

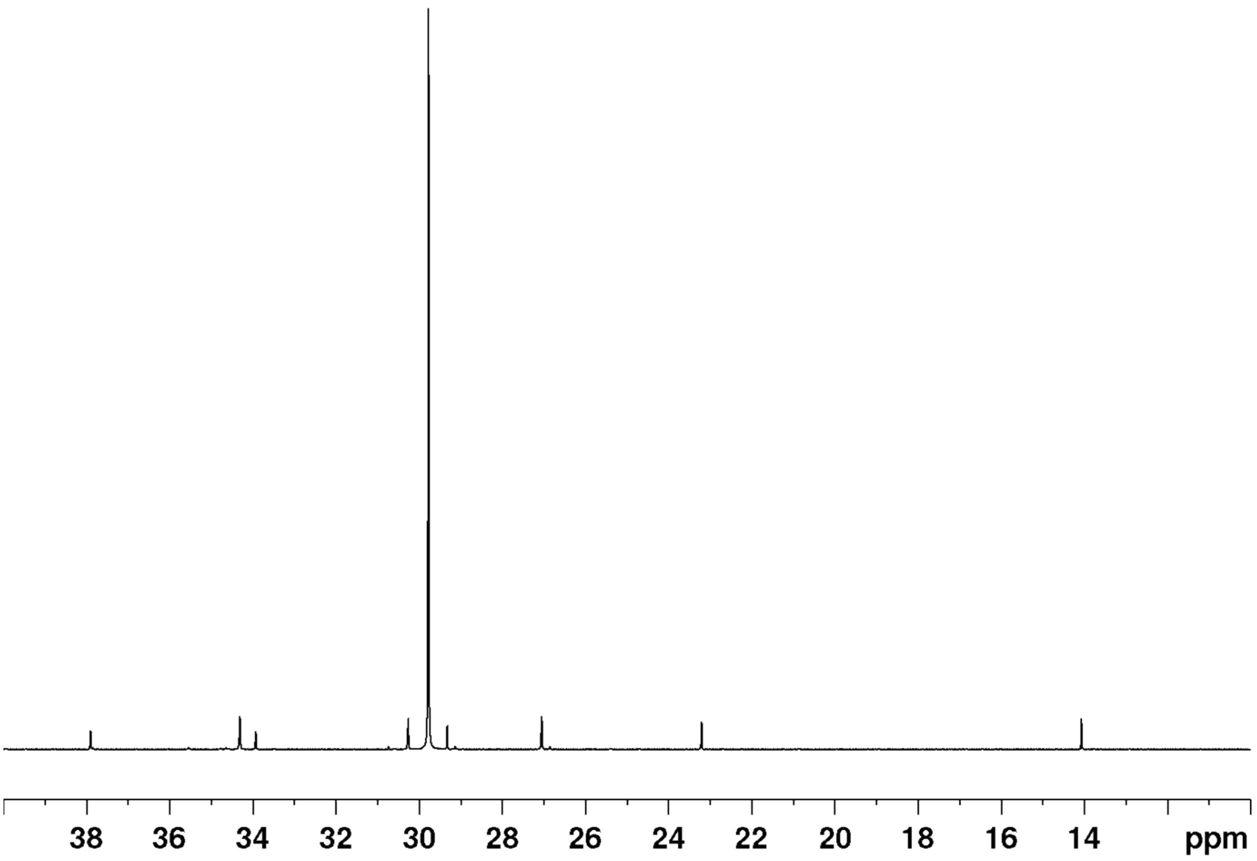

Fig. S24. $^{13}C\{^{1}H\}$ NMR spectrum (d_2-1,1,2,2-tetrachloroethane, 101 MHz, 393 K) of the polymers produced using $^{Me_2}SB(^{tBu}N,I^*)TiCl_2$ ($\mathbf{1_{sMAO}}$) with 0.2 M 1-hexene. Polymerisation conditions: 8.3 bar ethylene, 0.20 mg pre-catalyst ($[Al_{sMAO}]_0/[Ti]_0$ = 200), 5.0 mL heptane, 10 µmol TiBA and 80 °C. Reactions quenched at 5.5 bar ethylene uptake or after 60 minutes.

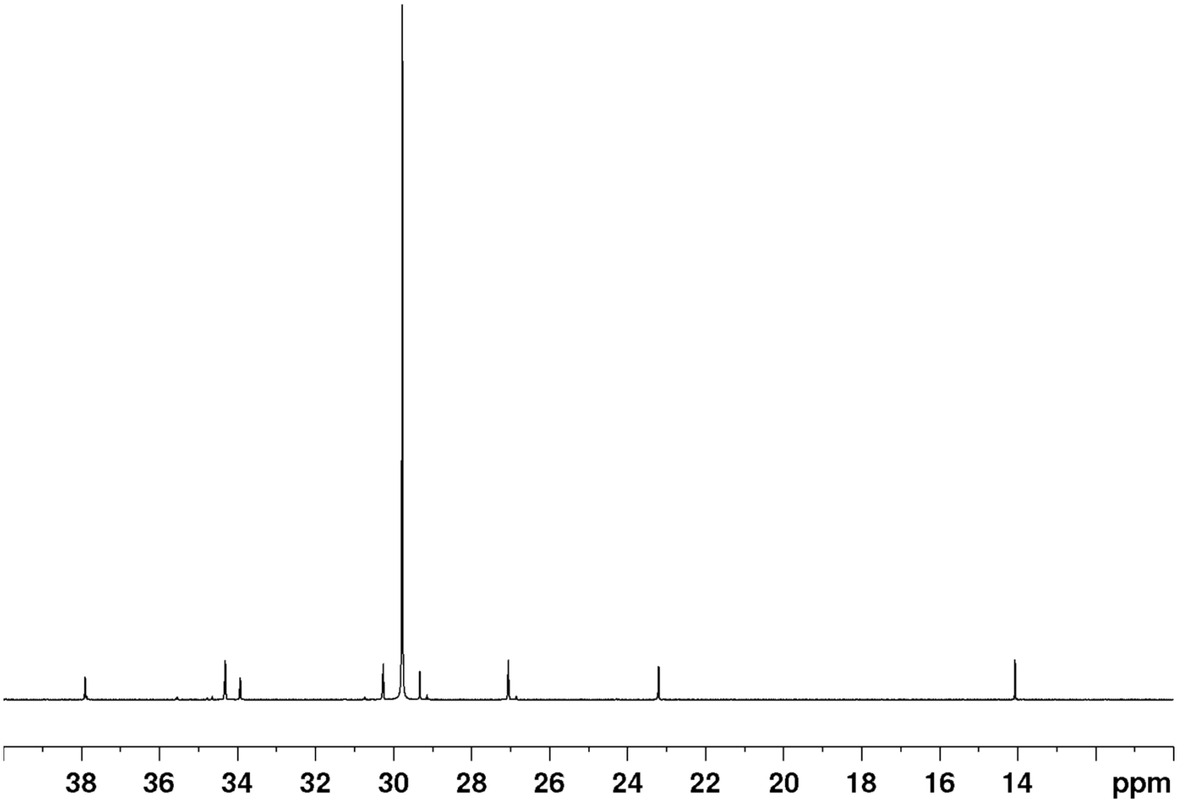

Fig. S25. $^{13}C\{^{1}H\}$ NMR spectrum (d_2-1,1,2,2-tetrachloroethane, 101 MHz, 393 K) of the polymers produced using $^{Me_2}SB(^{tBu}N,I^*)TiCl_2$ (**1_{sMAO}**) with 0.4 M 1-hexene. Polymerisation conditions: 8.3 bar ethylene, 0.20 mg pre-catalyst ($[Al_{sMAO}]_0/[Ti]_0$ = 200), 5.0 mL heptane, 10 μmol TiBA and 80 °C. Reactions quenched at 5.5 bar ethylene uptake or after 60 minutes.

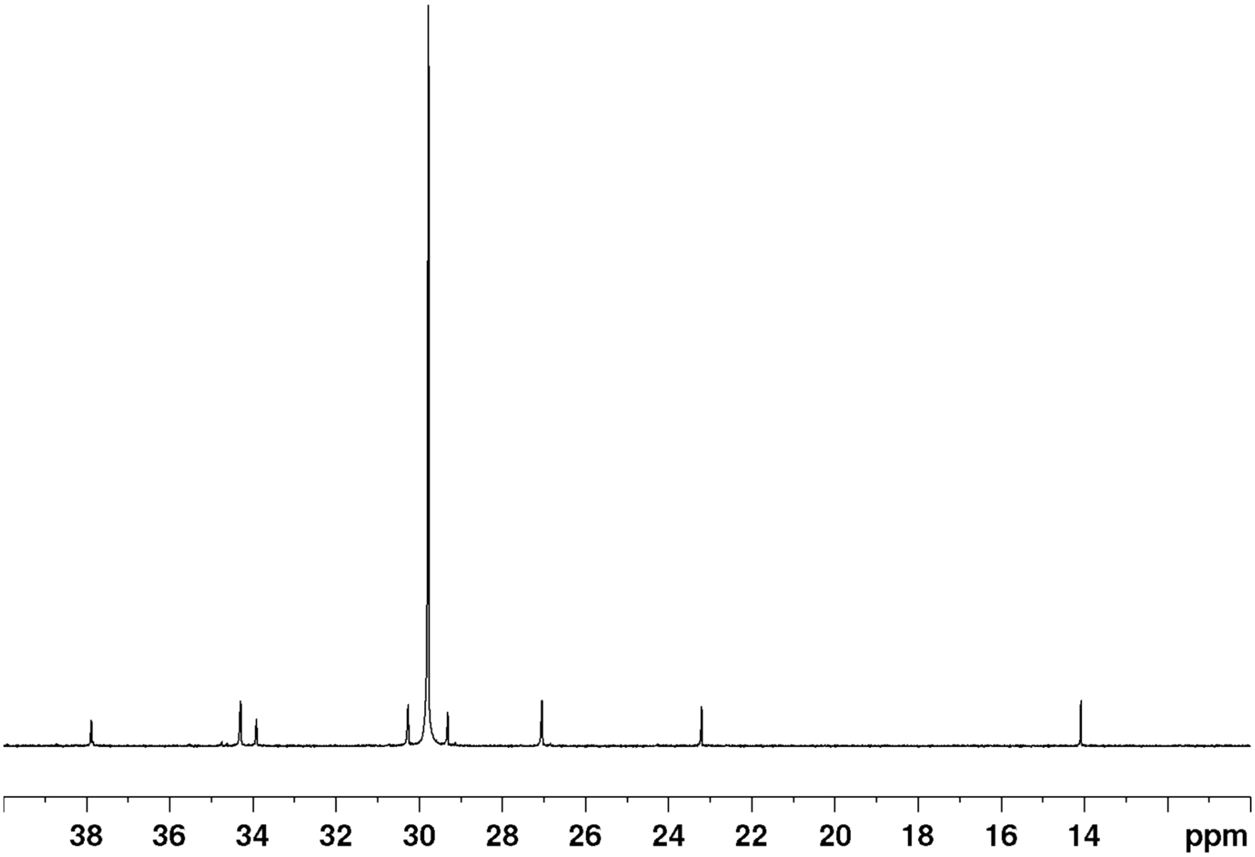

Fig. S26. $^{13}C\{^{1}H\}$ NMR spectrum (d_2-1,1,2,2-tetrachloroethane, 101 MHz, 393 K) of the polymers produced using $^{Me_2}SB(^{iPr}N,I^*)TiCl_2$ ($\mathbf{7_{sMAO}}$) with 0.2 M 1-hexene. Polymerisation conditions: 8.3 bar ethylene, 0.40 mg pre-catalyst ($[Al_{sMAO}]_0/[Ti]_0$ = 200), 5.0 mL heptane, 10 µmol TiBA and 80 °C. Reactions quenched at 5.5 bar ethylene uptake or after 60 minutes.

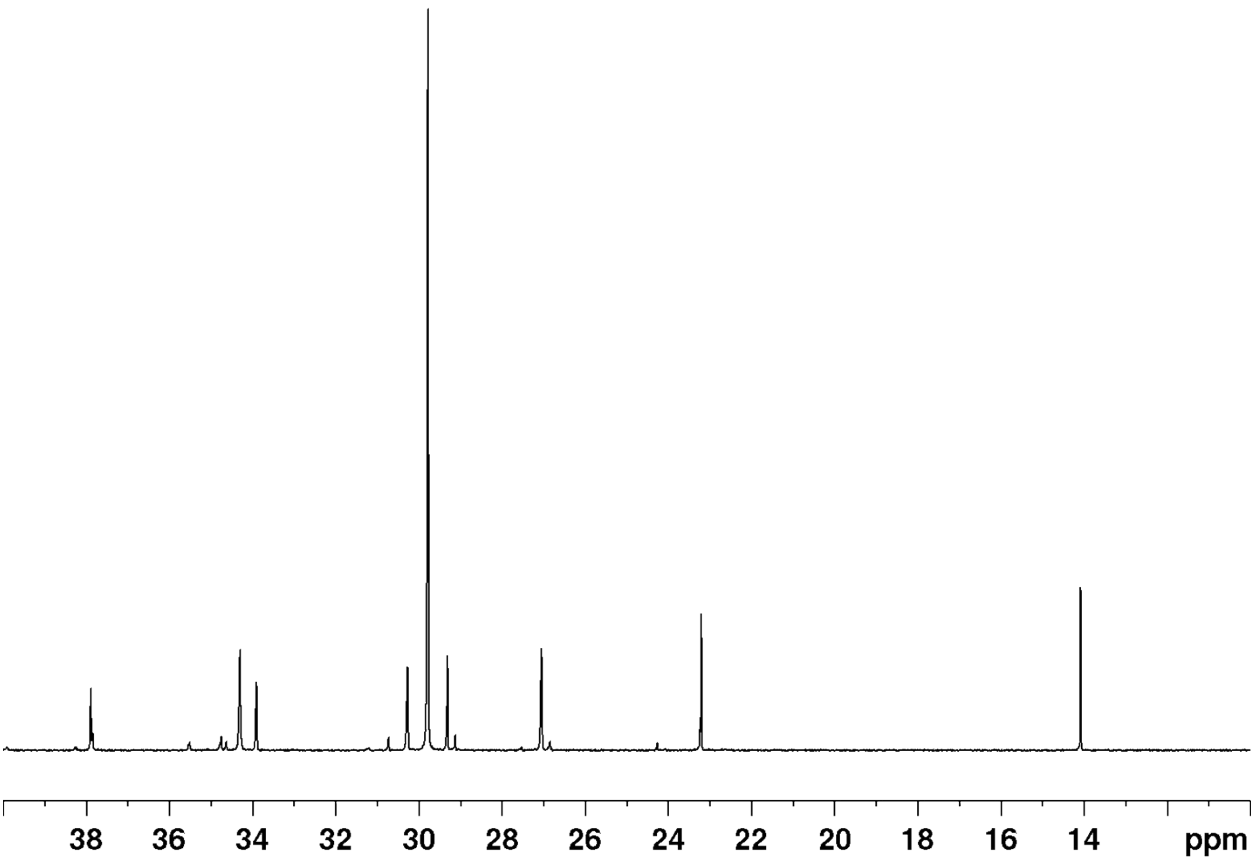

Fig. S27. $^{13}C\{^{1}H\}$ NMR spectrum (d_2-1,1,2,2-tetrachloroethane, 101 MHz, 393 K) of the polymers produced using $^{Me2}SB(^{iPr}N,I^{*})TiCl_2$ ($\mathbf{7_{sMAO}}$) with 0.4 M 1-hexene. Polymerisation conditions: 8.3 bar ethylene, 0.40 mg pre-catalyst ($[Al_{sMAO}]_0/[Ti]_0$ = 200), 5.0 mL heptane, 10 µmol TiBA and 80 °C. Reactions quenched at 5.5 bar ethylene uptake or after 60 minutes.

7. Added exemplar characterisation

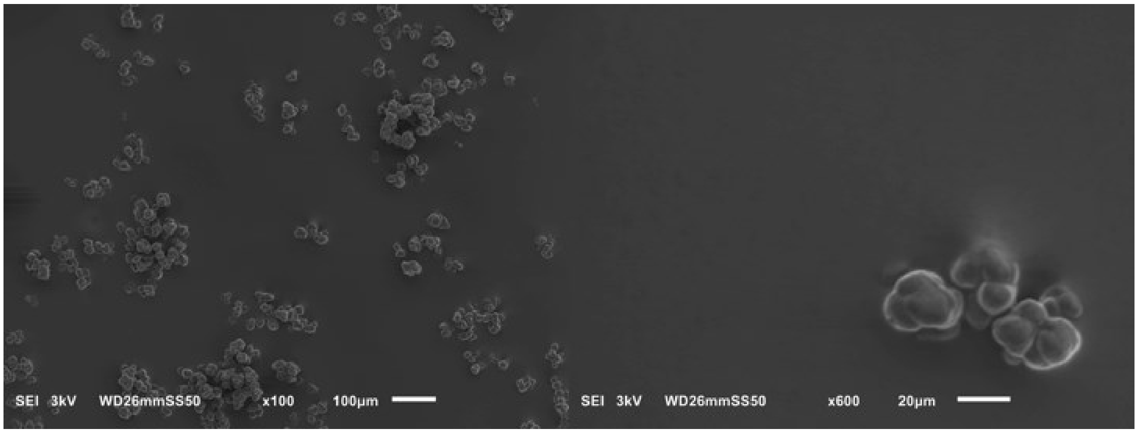

Fig. S28. SEM image of the polymer produced by sMAO-^{Me2}SB(^{tBu}N,I*)TiCl$_2$, $\mathbf{1}_{\mathbf{sMAO}}$, with 0.156 mL of 1-hexene; 10 mg catalyst ([Al]$_0$:[Ti]$_0$ = 200:1), 2 bar ethylene, 50 mL hexanes, 150 mg TiBA and 30 minutes.

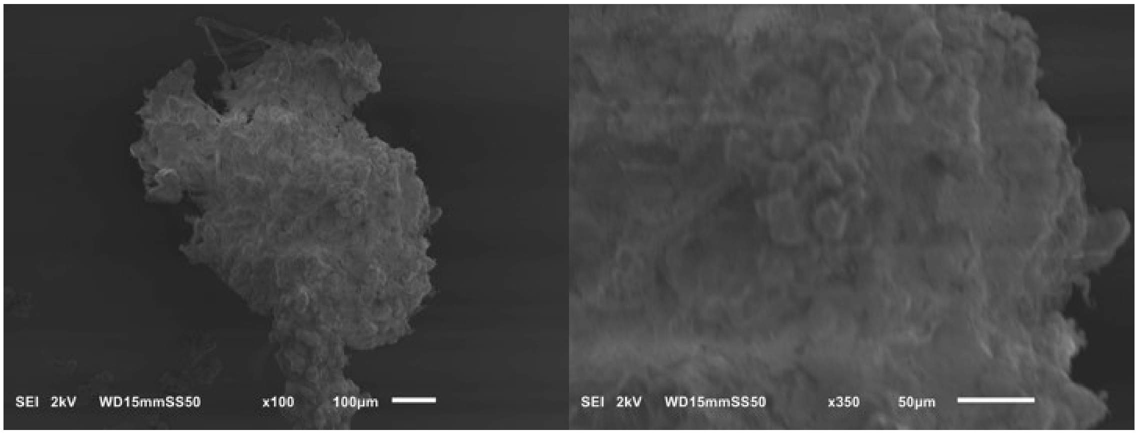

Fig. S29. SEM image of the polymer produced by sMAO-^{Me2}SB(^{tBu}N,I*)TiCl$_2$, $\mathbf{1}_{\mathbf{sMAO}}$, with 0.625 mL of 1-hexene; 10 mg catalyst ([Al]$_0$:[Ti]$_0$ = 200:1), 2 bar ethylene, 50 mL hexanes, 150 mg TiBA and 30 minutes.

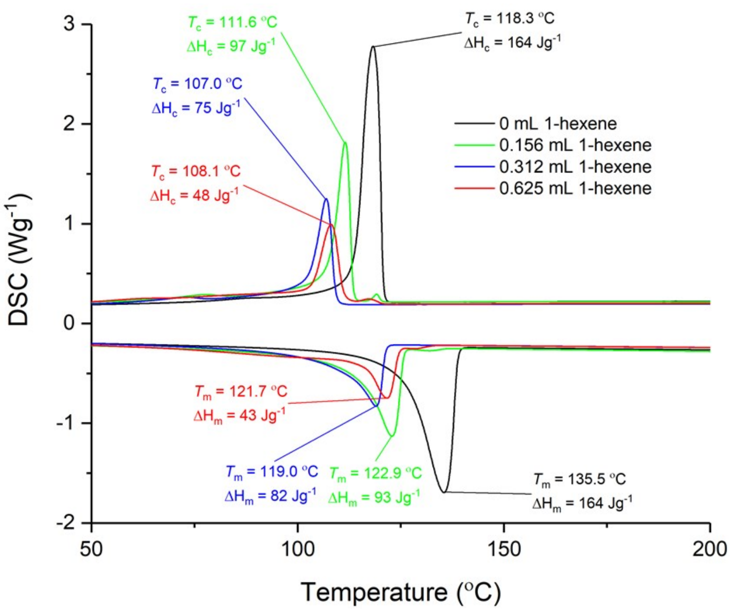

Fig. S30. DSC plot for polymers produced by sMAO-^{Me2}SB(^{tBu}N,I*)$TiCl_2$, $\mathbf{1}_{\mathbf{sMAO}}$, at 80 °C for 0 mL (black), 0.156 mL (green), 0.312 mL (blue), and 0.625 mL 1-hexene (red).

8. References

1. Williams, T. J.; Buffet, J.-C.; Turner, Z. R.; O'Hare, D., Group 4 permethylindenyl constrained geometry complexes for ethylene polymerisation catalysis. *Catal. Sci. Technol.* **2018,** *8* (21), 5454-5461.

2. Williams, T. J.; Smith, A. D. H.; Buffet, J.-C.; Turner, Z. R.; O'Hare, D., Group 4 constrained geometry complexes for olefin (co)polymerisation. *Mol. Catal.* **2020,** *486*, 110872.

3. Busico, V.; Cipullo, R.; Mingione, A.; Rongo, L., Accelerating the research approach to Ziegler–Natta catalysts. *Ind. Eng. Chem. Res.* **2016,** *55* (10), 2686-2695.
